# Bioinformatics-Driven mRNA-Based Vaccine Design for Controlling Tinea Cruris Induced by *Trichophyton rubrum*

**DOI:** 10.3390/pharmaceutics16080983

**Published:** 2024-07-25

**Authors:** Amir Elalouf, Hanan Maoz, Amit Yaniv Rosenfeld

**Affiliations:** Department of Management, Bar-Ilan University, Ramat Gan 5290002, Israel; hanan.maoz@gmail.com (H.M.); amityaro1@gmail.com (A.Y.R.)

**Keywords:** mRNA-based vaccine, *Trichophyton rubrum*, tinea cruris, bioinformatics

## Abstract

Tinea cruris, a dermatophyte fungal infection predominantly caused by *Trichophyton rubrum* and *Epidermophyton floccosum*, primarily affects the groin, pubic region, and adjacent thigh. Its recurrence is frequent, attributable to repeated fungal infections in susceptible individuals, especially those with onychomycosis or tinea pedis, which act as reservoirs for dermatophytes. Given the persistent nature of tinea cruris, vaccination emerges as a promising strategy for fungal infection management, offering targeted, durable protection against various fungal species. Vaccines stimulate both humoral and cell-mediated immunity and are administered prophylactically to prevent infections while minimizing the risk of antifungal resistance development. Developing fungal vaccines is challenging due to the thick fungal cell wall, similarities between fungal and human cells, antigenic variation, and evolutionary resemblance to animals, complicating non-toxic target identification and T-cell response variability. No prior research has shown an mRNA vaccine for *T. rubrum*. Hence, this study proposes a novel mRNA-based vaccine for tinea cruris, potentially offering long-term immunity and reducing reliance on antifungal medications. This study explores the complete proteome of *T. rubrum*, identifying potential protein candidates for vaccine development through reverse vaccinology. Immunogenic epitopes from these candidates were mapped and integrated into multitope vaccines and reverse translated to construct mRNA vaccines. Then, the mRNA was translated and computationally assessed for physicochemical, chemical, and immunological attributes. Notably, 1,3-beta-glucanosyltransferase, CFEM domain-containing protein, cell wall galactomannoprotein, and LysM domain-containing protein emerged as promising vaccine targets. Antigenic, immunogenic, non-toxic, and non-allergenic cytotoxic T lymphocyte, helper T lymphocyte, and B lymphocyte epitopes were selected and linked with appropriate linkers and Toll-like receptor (TLR) agonist adjuvants to formulate vaccine candidates targeting *T. rubrum*. The protein-based vaccines underwent reverse translation to construct the mRNA vaccines, which, after inoculation, were translated again by host ribosomes to work as potential components for triggering the immune response. After that, molecular docking, normal mode analysis, and molecular dynamic simulation confirmed strong binding affinities and stable complexes between vaccines and TLR receptors. Furthermore, immune simulations of vaccines with and without adjuvant demonstrated activation of immune responses, evidenced by elevated levels of IgG1, IgG2, IgM antibodies, cytokines, and interleukins. There was no significant change in antibody production between vaccines with and without adjuvants, but adjuvants are crucial for activating the innate immune response via TLRs. Although mRNA vaccines hold promise against fungal infections, further research is essential to assess their safety and efficacy. Experimental validation is crucial for evaluating their immunogenicity, effectiveness, and safety.

## 1. Introduction

Tinea cruris, also known as jock itch, is a dermatophyte fungal infection, most commonly caused by *Trichophyton rubrum* and *Epidermophyton floccosum,* that primarily affects the groin, pubic region, and adjacent thigh. Dermatophytes target keratinized tissues, including hair and the stratum corneum of the epidermis, leading to the manifestation of a distinct rash. Regions of skin overlap (intertriginous areas) provide conducive conditions for fungal colonization, facilitated by factors such as perspiration, moisture accumulation (maceration), and an alkaline pH, contributing to the heightened susceptibility of the groin region to fungal infections [1,2,3]. The infection is transmitted through fomites such as contaminated towels or hotel bedroom sheets or by autoinoculation from a reservoir on the hands or feet. The primary complications of tinea cruris are treatment failure and recurrence. These may occur due to several factors, including reinfection from close contacts, self-infection from other parts of the body, infection by less common fungal species, incorrect diagnosis, medication resistance, and failure to follow the prescribed treatment plan. Recurrence is common, as fungi may repeatedly infect susceptible individuals or those with onychomycosis or tinea pedis, which can serve as a dermatophyte reservoir [4,5,6,7].

The prevalence of tinea cruris is estimated to be around 20–25% of the general population globally. However, the prevalence can be higher in specific populations, such as athletes and individuals with lower socioeconomic status, diabetes mellitus, and improper hygiene, in certain conditions, such as high temperatures, increased humidity, excessive perspiration, and occlusive clothing, and in immunocompromised individuals. In addition, genetic factors can increase an individual’s susceptibility to dermatophytes [8,9]. In the United States, there have been approximately 29.4 million cases of superficial fungal infections (SFIs) and more than 51 million reported physician visits for such conditions [10]. Similarly, tinea corporis and tinea cruris (53.4%; 1682/3152) are Northern China’s most prevalent types of SFIs [11]. In a study conducted in Chitradurga, India, the prevalence of tinea cruris was 25% in the rural population. The study also found that tinea cruris was more prevalent in males (70%) than in females (30%) [12]. Tinea cruris is more common in men than women and affects adults more frequently than children [13]. 

The treatment options for tinea cruris include topical and systemic antifungal medications. Topical antifungal agents, such as clotrimazole, miconazole, terbinafine, and naftifine, are often used for localized infections [9,14,15,16,17]. These agents disrupt ergosterol synthesis, destabilizing fungal cell membranes and leading to cell death. Due to their clinical efficacy, topical azoles and allylamines are highly effective against localized infections. Oral antifungal medications (e.g., terbinafine, itraconazole, griseofulvin, or fluconazole) may be prescribed for severe or extensive infections. These agents target the fungal cell membrane or inhibit ergosterol synthesis, leading to cell death [18,19]. In addition to antifungal medications, good hygiene practices, such as wearing loose-fitting clothing made of cotton or moisture-wicking materials, washing affected skin areas daily, drying thoroughly, avoiding scratching, and washing clothes and bed linen frequently, are essential for preventing the spread of the infection and reducing the risk of recurrence [9]. In addition to traditional antifungal treatments, novel therapies like green biosynthesized silver nanoparticles using Achillea santolina extract have shown promise in treating dermatophytosis caused by *T. rubrum*. These nanoparticles have demonstrated significant antifungal activity against *T. rubrum*, inhibiting its growth and causing damage to fungal structures. This alternative therapy offers a potential treatment option with lower side effects compared to traditional antifungal drugs [20].

Due to the recurrence of tinea cruris, vaccines are beneficial for the treatment of fungal infections because they offer a more targeted, long-lasting, broad-range protection against multiple fungal species. They stimulate both humoral and cell-mediated immunity, are administered prophylactically to prevent fungal infections, and, most importantly, are less likely to lead to the development of antifungal resistance. This method contrasts with antifungal drugs, which typically target specific components of the fungal cell and can have side effects due to their broad-spectrum activity [21,22,23,24,25,26]. Literature indicates the promising recombinant subunit vaccines (NDV-3A for Candida [27]), peptide-based vaccines (a vaccine using peptide 10 (P-10) for Paracoccidioidomycosis [28]), antibody-based vaccines (monoclonal Mab28 antibodies against β-glucan particles [29]), and mRNA-based vaccines [21] for fungi. Although no fungal infection vaccine has been approved, two are undergoing clinical trials, with three reaching human clinical trials [30,31]. Science must overcome significant challenges before fungal vaccines can be licensed for human use. Clinical trials investigating adjuvant immunotherapies, with or without antifungal combinations, are crucial for further assessment [32].

Based on the literature search, there is no direct evidence of a vaccine being used as a treatment for *T. rubrum*. The search results mainly discuss the treatment of *T. rubrum* infections with antifungal medications such as terbinafine and itraconazole. However, the vaccine strain *T. mentagrophytes* F-01 has been developed and is 100% ready for mass production and sale. This vaccine strain and others like *T. verrucosum* F-02 are used to produce vaccines against Trichophyton infections in agricultural and carnivorous animals. The vaccine is administered twice at intervals of 14 days in a preventive dose to prevent the incidence of cattle Trichophyton infection. The highly immunogenic strain *T. mentagrophytes* F-01 is used to manufacture biological drugs against bovine dermatophytosis [33]. A living polyvalent vaccine has been developed to protect guinea pigs against challenge infection with virulent strains of dermatophytes, including *T. rubrum* [34]. Another study from the University of Georgia has developed a new vaccine that targets the three most common causes of fungal infections: Aspergillus, Candida, and Pneumocystis. The vaccine has shown broad, cross-protective antifungal immunity in animal models, making it a promising candidate for future clinical trials [35,36]. 

Developing antifungal vaccines presents several challenges, including the complexity and diversity of fungal pathogens, the lack of understanding of the immune response to fungal infections, and the high vaccine development and commercialization cost. However, bioinformatics approaches can help address some of these challenges by enabling the identification of potential vaccine targets based on the genomic sequence of the pathogen, predicting the immune response, and optimizing vaccine design. Despite these advances, there are still several challenges to overcome in the development of antifungal vaccines, including the need for better adjuvants and delivery systems, more effective and efficient vaccine production methods, and more clinical trials to evaluate the safety and efficacy of antifungal vaccines [25,37,38,39]. The emergence of new infectious diseases drives the need for innovative vaccine design strategies. Traditional methods, requiring pathogen cultivation and antigen identification, are time-consuming and expensive [40]. Reverse vaccinology, i.e., utilizing bioinformatics to identify antigens from genome sequences, accelerates vaccine development, particularly for multi-epitope vaccines. These vaccines, offering specificity, safety, and dual immune response induction, may benefit from protein-based adjuvants to enhance immunogenicity [41,42,43,44,45,46,47]. mRNA vaccines represent an innovative platform for expedited, safe, and tailored vaccine development against infectious diseases, encompassing viral outbreaks and emerging pathogens. Their significance stems from the capacity to utilize cellular machinery for antigen production devoid of infectious agents and genomic integration, eliciting robust humoral and cellular immune responses. This strategy affords multiple advantages over conventional vaccine platforms [48,49,50]. However, developing mRNA vaccines, while promising, requires further scientific evidence to ensure their efficacy and safety. Although recent successes have highlighted the potential of mRNA technology, these platforms are currently in stage II clinical trials and have not yet achieved full commercialization. This shortcoming underscores the necessity for continued rigorous testing and validation to address regulatory and manufacturing challenges and gain comprehensive scientific and regulatory confidence in mRNA vaccine platforms [48,51,52,53].

The development of vaccines against fungi is fraught with significant challenges, as highlighted extensively in the literature. One major hurdle is the thick cell wall of fungi, which impedes the penetration and accessibility of fungal antigens. Furthermore, the similarities between fungal and human cells complicate the identification of non-toxic drug targets. Additionally, antigenic variation within and between fungal species presents another layer of complexity. The evolutionary resemblance between the Fungi and Animalia kingdoms further complicates vaccine development. Moreover, the effectiveness of T-cell responses to fungal antigens may vary depending on the individual’s HLA haplotype. These factors underscore the intricate and multifaceted nature of developing effective and safe fungal vaccines [54,55,56,57]. No prior research has provided direct evidence of an mRNA-based vaccine specifically targeting *T. rubrum* for human use. While mRNA vaccines have garnered significant attention recently, especially for viral infections, their application to fungal pathogens, including *T. rubrum*, has not yet been investigated. An mRNA vaccine represents a promising alternative, potentially offering long-term immunity and reducing reliance on antifungal medications, often associated with side effects and the risk of resistance [58,59]. This study is the first to propose and design an mRNA-based vaccine to control tinea cruris caused by *T. rubrum*, presenting a novel preventive and therapeutic approach. This study first explores the entire proteome of *T. rubrum* and identifies promising potential protein candidates for vaccines. Subsequently, immunogenic epitopes derived from these candidates will be identified and compiled to design multitope vaccines via reverse vaccinology. Computational assessments will evaluate these vaccines’ physicochemical, chemical, and immunological attributes to identify a potential vaccine candidate for combating pathogenic *T. rubrum* and controlling tinea cruris.

## 2. Materials and Methods

### 2.1. Proteome Subtraction

Two reference proteomes of *T. rubrum* (UP000243015 and UP000008864) are available in the UniProt Proteome database. We selected the proteome with the highest number of coding protein sequences among these. Specifically, we utilized the *T. rubrum* strain ATCC MYA-4607/CBS 118892, corresponding to UniProt Proteome identifier UP000008864, which contains 10,006 coding protein sequences. Subsequently, membrane proteins were isolated from the *T. rubrum* proteome, and their antigenicity was assessed by employing the VaxiJen v2.0 server [60] (http://www.ddg-pharmfac.net/vaxijen/VaxiJen/VaxiJen.html, accessed on 30 May 2024) with a threshold of 0.5. Next, the DeepLoc 2.0 server [61] (https://services.healthtech.dtu.dk/services/DeepLoc-2.0/, accessed on 30 May 2024) was utilized to localize extracellular membrane proteins, which were further scrutinized for human homologs against the human proteome on NCBI using BLASTp; those sharing ≥35% identity were eliminated from consideration. The proteomic dataset of *T. rubrum* contains both redundant and non-redundant proteins. Redundant proteins, which may appear multiple times in the whole proteome, are less significant as strong vaccine candidates. Therefore, overlapping, duplicated, and unnecessary protein sequence entries were eliminated using the high-tolerance CD-HIT clustering database (http://weizhong-lab.ucsd.edu/cdhit-web-server/cgi-bin/index.cgi?cmd=cd-hit, accessed on 30 May 2024) to refine the selection of non-redundant immunogenic proteins [62,63]. Checking for the presence of transmembrane helices and estimation of protein molecular weights were conducted using TMHMM 2.0 [64] (https://services.healthtech.dtu.dk/services/TMHMM-2.0/, accessed on 30 May 2024) and Expasy tools [65] (https://web.expasy.org/protparam/, accessed on 30 May 2024), respectively. Proteins with a molecular weight <110 kDa and possessing ≤1 transmembrane helix were chosen for further evaluation [66,67]. Finally, conserved sequences among sorted proteins across various species within the genus Trichophyton were identified through BLASTP analysis and Multiple Sequence Alignment, ensuring that the designed chimeric vaccines exhibit cross-reactivity against pathogenic Trichophyton species.

### 2.2. Physiochemical Properties

The ExPASy-ProtParam and EMBOSS-PEPSTATS online webservers [68,69] calculated the physiochemical properties of the sorted proteins of *T. rubrum* to understand their biological functions, stability, structure, and interactions with other molecules. ExPASy-ProtParam calculates different physical and chemical parameters for protein sequences, including theoretical isoelectric point (pI), molecular weight in Dalton (Da), extinction coefficients, grand average of hydropathicity, aliphatic index, instability index, positively and negatively charged residues, and estimated half-life. SoluProt [70] was employed to predict the soluble protein expression in *E. coli*, where a solubility score above 0.5 indicates soluble expression, while a score below 0.5 indicates insoluble expression. EMBOSS-PEPSTATS calculates the absorption coefficients of protein in both reduced and cystine bridge form, isoelectric point, and probability of expression in inclusion bodies.

### 2.3. Profiling of T Cell and B Cell Epitopes and Features

The IEDB bioinformatics database tool [71] was employed to predict T and B cell (LBL) epitopes. Various prediction methods were utilized, including Ab initio, homology-based, LBL epitope, T cell epitope, and structure-based prediction.

#### 2.3.1. CTL Binding Epitope Screening and Profiling 

We employed the NetMHCpan EL 4.1 method [72] in cytotoxic T lymphocyte (CTL) binding epitope prediction server to forecast conserved CTL binding epitopes within sorted protein sequences of *T. rubrum*. Each predicted CTL epitope was further confirmed and identified for antigen-binding regions using the ProPred-I online server [73]. These epitopes were then assessed for antigenicity, toxicity, immunogenicity, and allergenicity using VaxiJen v2.0, ToxinPred2 [74], immunogenicity [75], and AllerTOP v2.0 [76] servers. 

#### 2.3.2. HTL Binding Epitope Screening and Profiling

The study predicted conserved helper T lymphocyte (HTL) binding epitopes within sorted proteins of *T. rubrum* using the IEDB recommended 2.22 method in the HTL binding prediction server [71]. All the predicted HTL binding epitopes were further confirmed using the ProPred online server [77]. Each epitope underwent assessment for allergenicity, antigenicity, IL10 inducing epitopes, IFN (Interferon)-Gamma inducing epitopes, interleukin (IL)-4 inducing epitopes, and toxicity through AllerTOP v2.0, VaxiJen v2.0, IL-10Pred [78], INFepitope [79], IL4Pred [80], and ToxinPred2, respectively.

#### 2.3.3. LBL Binding Epitope Screening and Profiling

Conserved LBL epitopes within sorted proteins of *T. rubrum* were forecasted by employing the BepiPred linear epitope prediction 2.0 method [81] in the antibody epitope prediction server. All the predicted LBL epitopes were further confirmed using the ABCpred server, which uses an artificial neural network (ANN) with 65.93% accuracy [82,83]. Subsequently, AllerTOP v2.0, ToxinPred2, and VaxiJen v2.0 servers were utilized to anticipate epitope allergenicity, toxicity, and antigenicity.

### 2.4. Epitope Conservancy Analysis

The recruited antigenic epitopes’ conservation within sorted proteins of *T. rubrum* was confirmed utilizing the Epitope Conservancy Analysis tool [84].

### 2.5. mRNA-Based Vaccine Construction, Its Structure Prediction and Characterization

The vaccines for sorted proteins of *T. rubrum* were constituted by linking antigenic epitopes of CTL, HTL, and LBL and an adjuvant together by AAY, EAAAK GPGPG, and KK linkers [85,86,87]. Each vaccine sequence began with a TLR4 agonist RS09 (APPHALS) adjuvant [88,89,90] and ended with a 6-His tag [91]. After that, we converted the protein-based vaccines to optimized mRNA vaccines by following the reported literature [92,93,94,95]. For the optimized vaccine expression, including efficient ribosome binding, transcription termination, and restriction enzyme cleavage sites, we utilized the JCAT online server for codon optimization [96]. For this purpose, we reverse translated the vaccines’ sequences. Further, we included 5′ m7GCap (7-methylguanylate cap), 5′ UTR (untranslated region), Kozak sequence, Signal peptide (tPA: tissue plasminogen activator) EAAAK linker at the N-terminal and MITD (major histocompatibility complex (MHC) I-targeting domain) sequence, stop codon, 3′ UTR, and poly (A) tail at the C-terminal to each vaccine construct. Subsequently, we employed the RNAfold web server [97,98,99] to predict the mRNA secondary structure thermodynamically and calculate the minimal free energy score. For the 3D model of the mRNA vaccines, the trRosettaRNA automated online server [100] (https://yanglab.qd.sdu.edu.cn/trRosettaRNA/, accessed on 30 May 2024) was utilized to build the models based on de novo folding, guided by deep learning restraints. 

mRNA vaccines enter immune cells, producing antigenic proteins that trigger adaptive immune responses. They also activate innate immunity, stimulate antibody production, and induce long-term cellular immunity, promising alternatives to traditional vaccines [49,101]. In this respect, each antigenic protein vaccine’s allergenicity, antigenicity, physicochemical characteristics, and toxicity were assessed via the AllerTop 2.0, VaxiJen 2.0, ANTIGENpro [102], ExPASy-ProtParam, EMBOSS-PEPSTATS, and Toxinpred2, respectively. SoluProt [70] was employed to predict the soluble protein expression in *E. coli*, where a solubility score above 0.5 indicates soluble expression, while a score below 0.5 indicates insoluble expression.

### 2.6. Secondary and Tertiary Structure Prediction, Refinement, and Verification

The secondary structure parameters of *T. rubrum* antigenic protein vaccine constructs were determined utilizing SOPMA [103]. The graphical representation was generated by PSIPRED [104]. Tertiary structure construction employed ColabFold [105], utilizing AlphaFold2 and Alphafold2-multimer, with sequence templates generated via HHsearch and MMseqs2. The 3D structures of each vaccine construct underwent refinement using GalaxyRefine [106], which rebuilds and repacks the amino acid residue side chains to relax the structure via molecular dynamics (MD) simulation. The refined structures were subsequently validated using PROCHECK [107], which analyzes residue-by-residue and overall structure geometry, constructing the Ramachandran Plot. 

### 2.7. Prediction of Continuous and Discontinuous B Cell Epitopes

The IEDB server’s ElliPro tool [108] represented each vaccine construct’s continuous and discontinuous B cell epitopes.

### 2.8. Molecular Docking

Immune response against fungus has been reported to be supported by both TLR2 and TLR4 interactions [109,110,111]. The ClusPro v2.0 web server (https://cluspro.bu.edu/home.php, accessed on 2 April 2024) was used to dock the TLR2 (PDB ID: 6NIG) and TLR4 (PDB ID: 4G8A) proteins with the vaccines individually [112,113,114,115]. All unnecessary ligands and heteroatoms were removed from the TLR2 and TLR4 and uploaded to the ClusPro 2.0 server with all the constructed vaccines as a separated ligand for protein–protein docking. The highest-ranked model for each docking prediction was retrieved and evaluated with PRODIGY [116,117] (https://wenmr.science.uu.nl/prodigy/, accessed on 2 April 2024). PRODIGY assessed the binding affinity, dissociation constant, and the number of contacts created between the vaccines and both TLR2 and TLR4 receptors at 37 °C (protein–protein complexes). Additionally, PDBsum was used to obtain a graphical illustration with additional features like interface residues with area, salt bridges, hydrogen bonds, and non-bonded contacts of the interactions between vaccines and receptors [118].

### 2.9. Normal Mode Analysis

The iMODS online server [119] (http://imods.chaconlab.org/, accessed on 2 April 2024) was employed to determine normal mode analysis (NMA) of collective motion in internal coordinates and torsional angles and protein flexibility following molecular docking of the best-docked vaccine–TLR complexes. Essential dynamics were utilized for protein stability and motion prediction based on various factors. The basic interface atomic model was used with the CA option for the Coarse Grain model representations to account for alpha carbon (Cα) atoms for whole residue mass.

### 2.10. Molecular Dynamic Simulation

The software application Desmond (https://www.schrodinger.com/platform/products/desmond/, accessed on 15 June 2024) from Schrödinger LLC (New York, NY, USA) was employed to conduct 100 ns (100,000 ps) of MD simulations. For this purpose, rigid binding assessments of the vaccines’ potential interactions with the TLRs were performed using protein–protein docking in MD simulations. Newton’s classical equation of motion was applied in the MD simulations to predict the protein–protein binding status in the physiological environment [120,121,122,123,124,125]. The selected vaccine–receptor interactions from the docking experiments were optimized and minimized using Maestro’s Protein Preparation Wizard, ensuring there were no steric conflicts, poor contacts, or distorted geometries. The systems were built with the System Builder tool, employing the TIP3P (Intermolecular Interaction Potential 3 Points Transferable) as the solvent model in an orthorhombic box with the OPLS_2005 force field [126,127]. During the simulation, conditions were set at 300 K temperature and 1 atm pressure to mimic physiological environments, with counter ions added for model neutralization and 0.15 M sodium chloride included. Trajectories were recorded every 100 ps for analysis, and the stability of vaccine–receptor interactions was assessed by measuring the Root Mean Square Deviation (RMSD) over time [120,121,122,123,124,125].

### 2.11. Immune Simulation of Vaccine Constructs

The C-ImmSim (https://kraken.iac.rm.cnr.it/C-IMMSIM/, accessed on 2 April 2024) [128], an online antigen-based immune simulator, was utilized to evaluate the vaccine constructs with and without adjuvant for immunogenic profiles. This web server predicts immune reactions by hybridization, combining the position-specific scoring matrix (PSSM) with machine learning algorithms. Each vaccine received three doses of 1000 antigens, with an 8-week interval between doses. The doses were administered at time-steps 168 and 504 (equivalent to 8 h in real life), respectively, with the first dose given at time-step 1. The simulation was conducted for 1050 time-steps using default parameters.

## 3. Results

### 3.1. Protein Selection

From the proteomic analysis of *T. rubrum*, four proteins (1,3-beta-glucanosyltransferase, CFEM domain-containing protein, cell wall galactomannoprotein, and LysM domain-containing protein) were identified based on meeting specific criteria, including antigenicity (≥0.5), extracellular localization (≥0.612), ≤35% human homolog identity, ≤1 transmembrane helix, and a molecular weight of less than 110 kDa. These findings are summarized in Table 1. These proteins play pivotal roles in fungal biology, including cell wall biosynthesis, pathogenesis, virulence, and interactions with the host immune system.

### 3.2. Physiochemical Properties

The systematic examination of the physiochemical properties of proteins—1,3-beta-glucanosyltransferase, CFEM domain-containing protein, cell wall galactomannoprotein, and LysM domain-containing protein—of *T. rubrum* provides crucial insights into their functionality within biological contexts. In designing vaccines against *T. rubrum* fungal infections, the physiochemical properties of sorted proteins reveal a mildly acidic nature with an abundance of negatively charged amino acid residues; notably, 1,3-beta-glucanosyltransferase and LysM domain-containing protein exhibit higher stability than other proteins. Table 2 presents a comprehensive overview of the physiochemical characteristics of sorted proteins of *T. rubrum*.

### 3.3. T Cell and B Cell Epitope and Feature Profiling

An IDEB server was utilized to predict binding epitopes of 1,3-beta-glucanosyltransferase, CFEM domain-containing protein, cell wall galactomannoprotein, and LysM domain-containing protein from *T. rubrum* for CTL, HTL, and LBL.

#### 3.3.1. CTL Binding Epitope Prediction

The CTL binding epitopes of 1,3-beta-glucanosyltransferase, CFEM domain-containing protein, cell wall galactomannoprotein, and LysM domain-containing protein sequences of *T. rubrum* were predicted by IEDB and confirmed for antigen binding regions by ProPred-I online servers. Table 3 displays the filtered CTL binding epitopes selected based on their antigenicity, non-allergenicity, immunogenicity, and non-toxic properties of sorted proteins. 

#### 3.3.2. HTL Binding Epitope Prediction

HTL binding epitopes of 1,3-beta-glucanosyltransferase, CFEM domain-containing protein, cell wall galactomannoprotein, and LysM domain-containing protein sequences of *T. rubrum* were predicted by IEDB and confirmed by ProPred. The antigenicity, IFN-gamma-inducing, IL4-inducing, and IL10-inducing effects, non-allergenicity, and non-toxicity of all the epitopes were assessed for utilization in vaccine construction, as shown in Table 4.

#### 3.3.3. LBL Binding Epitope Prediction

The LBL epitopes of 1,3-beta-glucanosyltransferase, CFEM domain-containing protein, cell wall galactomannoprotein, and LysM domain-containing protein sequences of *T. rubrum* were predicted by IEDB and confirmed by ABCpred server. The predicted LBL binding epitopes were screened based on their antigenicity, non-allergenicity, and non-toxic properties and are presented in Table 5.

### 3.4. Epitope Conservancy Analysis

All the recruited CTL, HTL, and LBL epitopes selected for vaccine construction exhibited complete conservation, as determined by epitope conservancy analysis.

### 3.5. mRNA-Based Vaccine Construction and Characterization

A systematic approach was employed for developing protein-based vaccines targeting 1,3-beta-glucanosyltransferase, CFEM domain-containing protein, cell wall galactomannoprotein, and LysM domain-containing protein of *T. rubrum* [42,129]. Antigenic, immunogenic, non-toxic, interleukin-inducing, and non-allergenic CTL, HTL, and LBL epitopes were arranged. These epitopes were connected using AAY, GPGPG, KK, and EAAAK linkers. As per prior methodologies, a TLR4 agonist RS09 (APPHALS) adjuvant [88,89,90] was attached at the N-terminal and a 6  ×  His tag at the C-terminus. Epitopes that exhibited ≥70% similarity in amino acid composition were merged into a singular epitope. 

Further, the mRNA vaccine construct for BGTV was 5′ m7GCap–5′ UTR–Kozak sequence–Signal peptide (tPA)–EAAAK linker- Adjuvant (RS09)—EAAAK Linker–NEVQPRMFTEVQALYGDKM–KK Linker–TSADNSYQDPLADVKS–AAY Linker–SNGTEFFMK–GPGPG Linker–SYQDPLADV–GPGPG Linker–QELQTNTIRV–GPGPG Linker–YTRYTSVID–GPGPG Linker-YTNVIGFFAG–GPGPG Linker–FWGYNIYSW–GPGPG Linker–NFNVPVFFA–GPGPG–MITD sequence–Stop codon–3′ UTR–Poly (A) tail. For CDPV, it was 5′ m7GCap–5′ UTR–Kozak sequence–Signal peptide (tPA)–EAAAK linker-Adjuvant (RS09)–EAAAK Linker-CSNADFQHGLRDCTHEACPGEKVEQVVQAGLQACREMGGAPGSSTGAPTTGTGSGTTTGTPTSGSGSETTAPSTSGSGSAPAPTSGGHSTPYSTIPAGPTVITSGTHVVTTSRPPTTLYTEVSGSQTGSESSSPTGTGSESTSAPETTSPSSTEGGSSPSSTEGSGNGGSGGSETSGSGNGPSQTPSQGIAPKATGLGV–KK Linker–VVTTSRPPTTLYTEVSGSQT–AAY Linker–TSGSGNGPSQTPSQGGIAP–AAY Linker–THVVTTSRPPTTLYTEVSGS–GPGPG Linker–SSSPTGTGSES–GPGPG Linker–PSSTEGGSS–GPGPG–MITD sequence–Stop codon–3′ UTR–Poly (A) tail.

For GMPV, it was 5′ m7GCap–5′ UTR–Kozak sequence–Signal peptide (tPA)–EAAAK linker- Adjuvant (RS09)– EAAAK Linker-PSTFSSVPEAIGDLDPISASIEGLSQRIAQSPGGITELMS–KK Linker–LSQRIAQSPGGITEL–AAY Linker–ATSTKVPLIKAVPGG–AAY Linker–AQSPGGITE–GPGPG Linker–MSVTNDIYD–GPGPG–MITD sequence–Stop codon–3′ UTR–Poly (A) tail. Lastly, for LDPV, it was 5′ m7GCap–5′ UTR–Kozak sequence–Signal peptide (tPA)–EAAAK linker- Adjuvant (RS09)–EAAAK Linker-GATISTSMPMPTPSGPQPQMPGIVSNC–KK Linker–TTTRAMTTTISSDAP–AAY Linker–SIQTKYGISTDQFKAWNPYINAE–AAY Linker–PSTTTTAKP–GPGPG Linker–TRAMTTTIS–GPGPG–MITD sequence–Stop codon–3′ UTR–Poly (A) tail.

**Figure 1:**
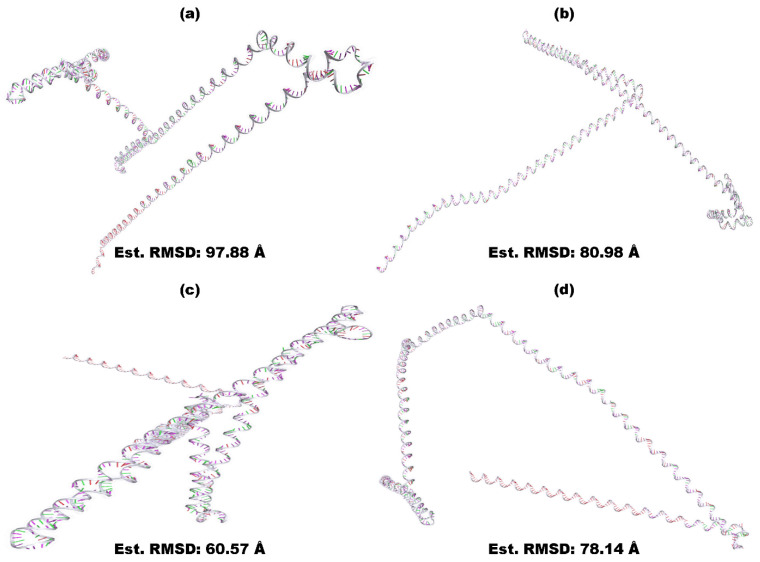
Predicted 3D structures of mRNA-derived vaccine candidates BGTV (**a**), CDPV (**b**), GMPV (**c**), and LDPV (**d**). Generated using trRosettaRNA.

The amino acid-based vaccines underwent reverse translation, and the resulting optimized nucleotide sequences of the constructed BGTV, CDPV, GMPV, and LDPV candidates are provided in Supplementary Table S1. The optimal secondary structures of mRNA BGTV, CDPV, GMPV, and LDPV candidates exhibited minimal free energies of −272.60 kcal/mol, −416.20 kcal/mol, −217.30 kcal/mol, and −185.10 kcal/mol, respectively, as illustrated in Supplementary Figure S1. Additionally, the thermodynamic free energies of the BGTV, CDPV, GMPV, and LDPV candidates were determined to be −286.80 kcal/mol, −442.11 kcal/mol, −233.14 kcal/mol, and −201.13 kcal/mol, respectively. The 3D structures of mRNA-derived BGTV, CDPV, GMPV, and LDPV candidates, predicted using trRosettaRNA, are presented in Figure 1.

When mRNA vaccines enter host immune cells, these are translated by the host protein factory, i.e., ribosomes, into antigenic proteins that trigger adaptive immune responses. They also activate innate immunity, stimulate antibody production, and induce long-term cellular immunity, promising alternatives to traditional vaccines [49,101]. The molecular characteristics of translated protein-based vaccines, i.e., BGTV (1,3-beta-glucanosyltransferase targeting vaccine), CDPV (CFEM domain-containing protein targeting vaccine), GMPV (cell wall galactomannoprotein targeting vaccine), and LDPV (LysM domain-containing protein targeting vaccine), exhibit unique attributes tailored to their specific target (as shown in Table 6). For instance, the BGTV, comprising 158 amino acids, demonstrates stability, with an instability index of 15.64 and a negative GRAVY score (−0.564), indicating hydrophilicity. In contrast, the CDPV, with 319 amino acids, shows signs of instability, reflected in its high instability index (50.49) and negative GRAVY score (−0.653). The GMPV, consisting of 124 amino acids, similarly exhibits instability characteristics, as evidenced by its high instability index (66.81) and relatively low solubility (0.435). Conversely, the LDPV, comprising 119 amino acids, displays stability, with a lower instability index (31.74) and comparable solubility (0.294) to the GMPV vaccine. These diverse molecular profiles highlight the importance of considering specific protein targets and optimizing vaccine design to enhance stability, immunogenicity, and safety for effective immunotherapy.

### 3.6. Secondary and Tertiary Structure Modeling, Refinement, and Verification

**Figure 2:**
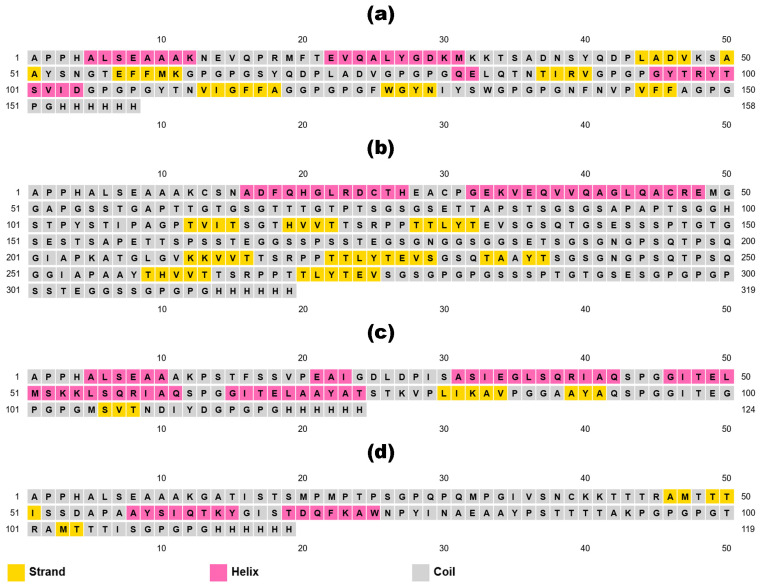
Visual representation of secondary structures of BGTV (**a**), CDPV (**b**), GMPV (**c**), and LDPV (**d**).

The secondary structures of constructed vaccines predicted from the SOPMA server reveal distinct distributions of alpha helix, extended strand, beta-turn, and random coil elements. For the BGTV, alpha helices constitute the highest proportion at 28.48%, followed by random coil, at 46.84%. In contrast, the CDPV displays a higher prevalence of random coil (72.10%) and a relatively lower percentage of alpha helices (11.29%). The GMPV exhibits alpha helices (29.03%) and extended strands (8.06%). Conversely, the LDPV shows a significant proportion of extended strands (25.21%) and random coils (61.34%). These variations in secondary structure compositions across the target proteins underscore the diverse structural characteristics vaccine designs need to consider for optimal immunogenicity and efficacy (Figure 2).

**Figure 3:**
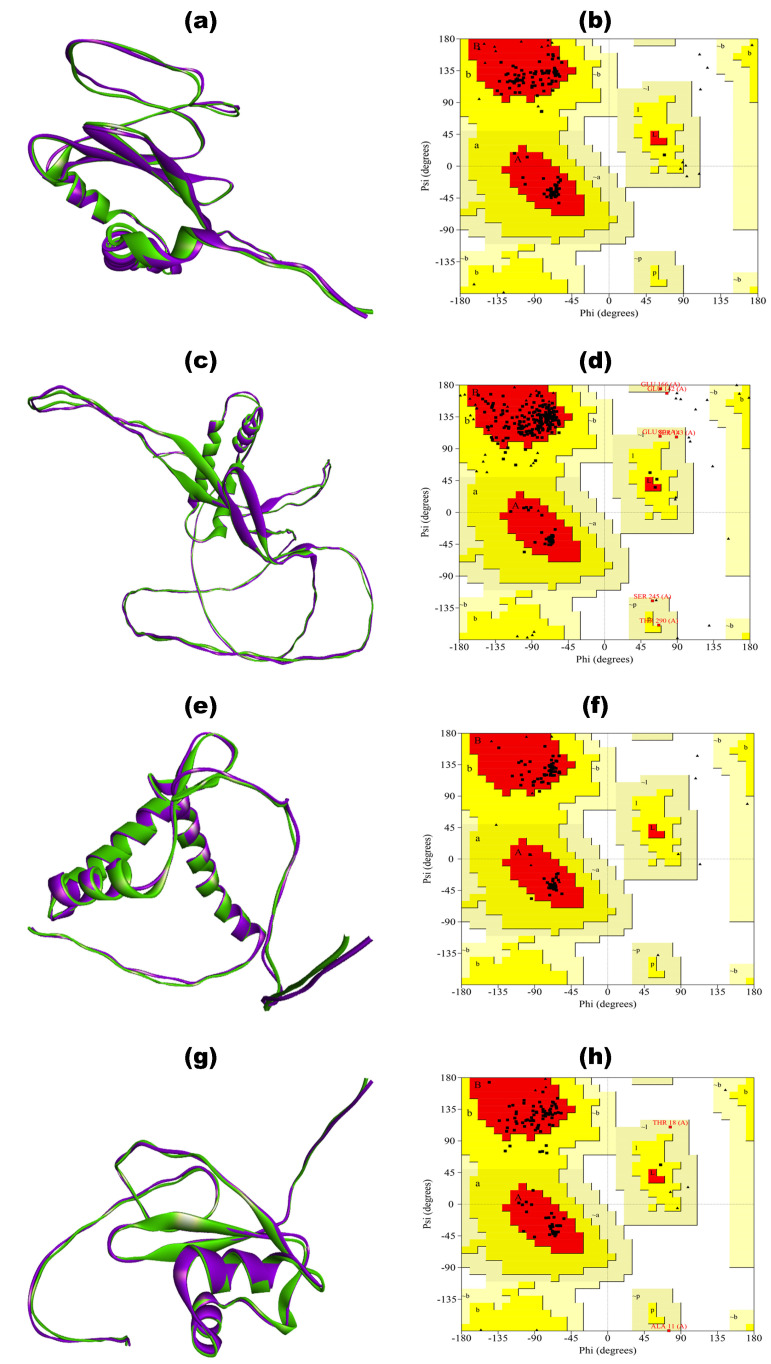
Superimposed 3D models of unrefined (purple) and refined (green) BGTV (**a**), CDPV (**c**), GMPV (**e**), and LDPV (**g**) with Ramachandran plots of refined 3D constructs of BGTV (**b**), CDPV (**d**), GMPV (**f**), and LDPV (**h**).

The tertiary structures of all the vaccine constructs (BGTV, CDPV, GMPV, and LDPV) were built using ColabFold (represented in Figure 3, purple), which uses a subset of the MSA as input to the model, with Alphafold2 subsampling the MSA to a maximum of 512 cluster centers and 1024 extra sequences [105]. All the modeled vaccines were validated through a Ramachandran plot. The plot results confirmed that the predicted structures were not of good quality because of less than 90% of residues in favored regions, as shown in Supplementary Figure S2 and Table 7. For instance, BGTV has only 60.9% residues in the most favored regions of the plot, while CDPV, GMPV, and LDPV have 30.8%, 57.6%, and 42.9%, respectively. Since then, the refinement of 3D vaccine structures has been required to obtain good-quality models. 

The GalaxyRefine online server was employed to conduct structural relaxation of 3D-modeled constructs of BGTV, CDPV, GMPV, and LDPV. This process yielded five refined structures for each vaccine construct. Subsequently, the structure exhibiting the highest Rama-favored value and the lowest Root Mean Square Deviation (RMSD) value (as delineated in Supplementary Table S2) was chosen for subsequent analysis. The refined 3D vaccine constructs (green) superimposed on the unrefined 3D vaccine constructs (purple) are exhibited in Figure 3. Ramachandran plots of all the refined vaccine constructs confirmed good quality models having over 90% in the amino acid residues in the most favored regions (as shown in Figure 3 and Table 7).

### 3.7. Discontinuous and Continuous B Cell Epitope Prediction

The folding process of the vaccine model proteins leads to the emergence of conformational or discontinuous B-cell epitopes. The ElliPro server analysis revealed the presence of seven linear epitopes and ten discontinuous B-cell epitopes within BGTV. Similarly, CDPV exhibited four linear and six discontinuous B-cell epitopes, while GMPV demonstrated five linear and five discontinuous B-cell epitopes. Moreover, LDPV showcased six linear and nine discontinuous B-cell epitopes. Supplementary Figure S3 illustrates the spatial distribution of continuous and discontinuous B-cell epitopes across BGTV, CDPV, GMPV, and LDPV.

### 3.8. Molecular Docking

Following vaccine administration, its primary objective is to initiate the immune response against the foreign antigen. Toll-like receptors (TLRs) play a pivotal role in recognizing molecules from pathogens and activating innate immunity. Among TLRs involved in fungal recognition, TLR2 and TLR4 are prominent. TLR2 forms complexes with TLR1 or TLR6 to detect fungal cell wall components like mannoprotein, while TLR4 recognizes fungal mannans and β-glucans. Additionally, TLR2 and TLR4 collaborate with receptors like Dectin-1 to bolster the immune response against fungi. Activation of TLRs triggers macrophages, neutrophils, and dendritic cells to produce inflammatory cytokines and eliminate fungi. Moreover, TLRs influence adaptive immunity by promoting Th1 or Th17 responses [42,130,131,132].

**Figure 4:**
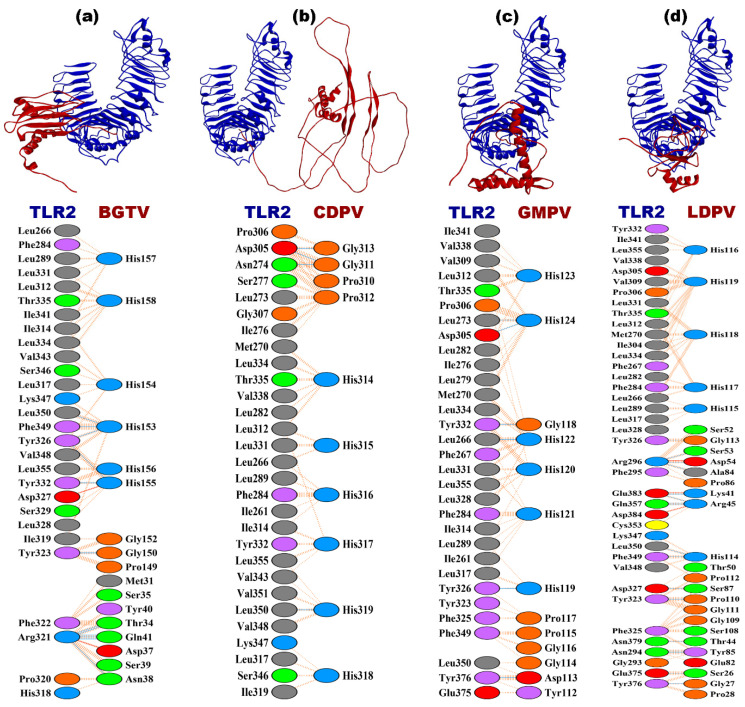
Docking complexes of vaccine candidates (red) against *T. rubrum* and TLR2 receptor (blue). (**a**) BGTV-TLR2; (**b**) CDPV-TLR2; (**c**) GMPV-TLR2; (**d**) LDPV-TLR2.

The docking complexes BGTV-TLR2, CDPV-TLR2, GMPV-TLR2, and LDPV-TLR2 exhibit distinct free energy changes (dG) for the binding range from −12.7 to −15.1 kcal mol^−1^, suggesting favorable binding energies across these complexes along with their dissociation constants (Kd) at the 25 °C span and indicating strong binding affinities (as shown in Table 8). Analyzing the binding affinity in terms of the lowest energy states, LDPV-TLR2 demonstrates the highest binding affinity, with energies of −1453.9 kcal mol^−1^. These binding affinities predominantly stem from electrostatic, hydrophobic, Van der Waals, and electrostatic interactions with salt bridges, hydrogen bonds, and non-bonded contacts that play significant roles in binding. For instance, CDPV-TLR2 forms the highest number of salt bridges (4), whereas BGTV-TLR2 forms the fewest (2). Regarding hydrogen bonds, LDPV-TLR2 forms the highest number (17), whereas BGTV-TLR4 forms the fewest (14). The number of non-bonded contacts ranges from 150 to 215, with LDPV-TLR2 demonstrating the highest count and BGTV-TLR4 the lowest (as shown in Figure 4 and Table 8).

**Figure 5:**
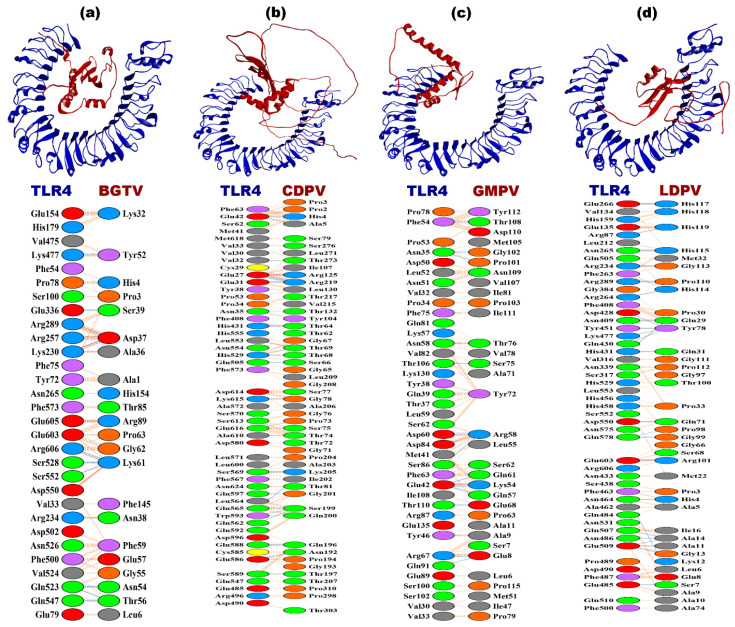
Docking complexes of vaccine candidates (red) against *T. rubrum* and TLR4 receptor (blue). (**a**) BGTV-TLR4; (**b**) CDPV-TLR4; (**c**) GMPV-TLR4; (**d**) LDPV-TLR4.

The docking complexes involving TLR4 (BGTV, CDPV, GMPV, and LDPV) exhibit similar trends in interface residues, interface areas, dG values, and Kd values compared to their TLR2 counterparts. However, notable differences exist in the calculated binding affinities and intermolecular interactions. For example, LDPV-TLR4 demonstrates the highest binding affinity, with a dG of −20.4 kcal mol^−1^, and the lowest dissociation constant of 1.1 × 10^−15^ M. Salt bridges, hydrogen bonds, and non-bonded contacts also vary across the TLR4 complexes, reflecting unique interaction profiles within this receptor system (as shown in Figure 5 and Table 8).

### 3.9. Normal Mode Analysis

**Figure 6:**
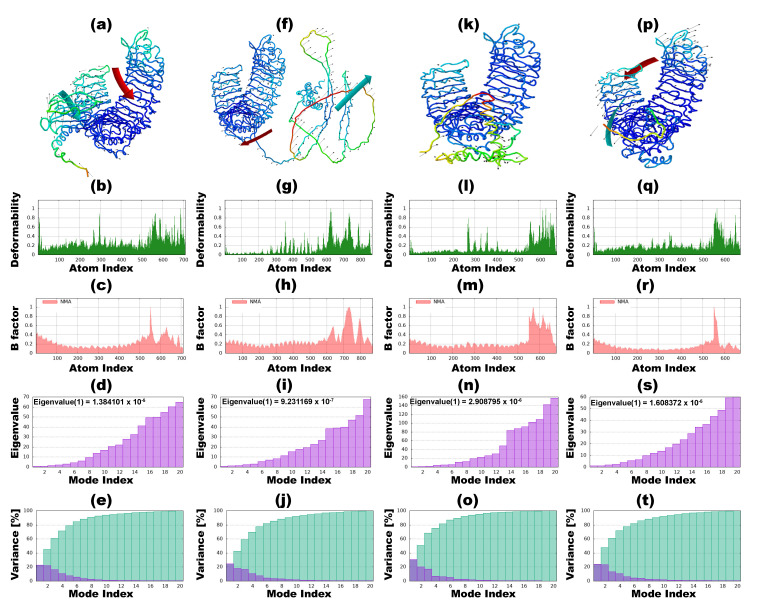
Normal mode analysis (NMA) of vaccine candidates against *T. rubrum* and TLR2 receptor complexes by iMODs. (**a**–**e**) iMODS results of BGTV-TLR2 complex. (**a**) NMA mobility; (**b**) main-chain deformability; (**c**) B-factor values; (**d**) the eigenvalue; (**e**) variance. (**f**–**j**) iMODS results of CDPV-TLR2 complex. (**f**) NMA mobility; (**g**) main-chain deformability; (**h**) B-factor values; (**i**) the eigenvalue; (**j**) variance; (**k**–**o**) iMODS results of GMPV-TLR2 complex. (**k**) NMA mobility; (**l**) main-chain deformability; (**m**) B-factor values; (**n**) the eigenvalue; (**o**) variance; (**p**–**t**) iMODS results of LDPV-TLR2 complex. (**p**) NMA mobility; (**q**) main-chain deformability; (**r**) B-factor values; (**s**) the eigenvalue; (**t**) variance.

**Figure 7:**
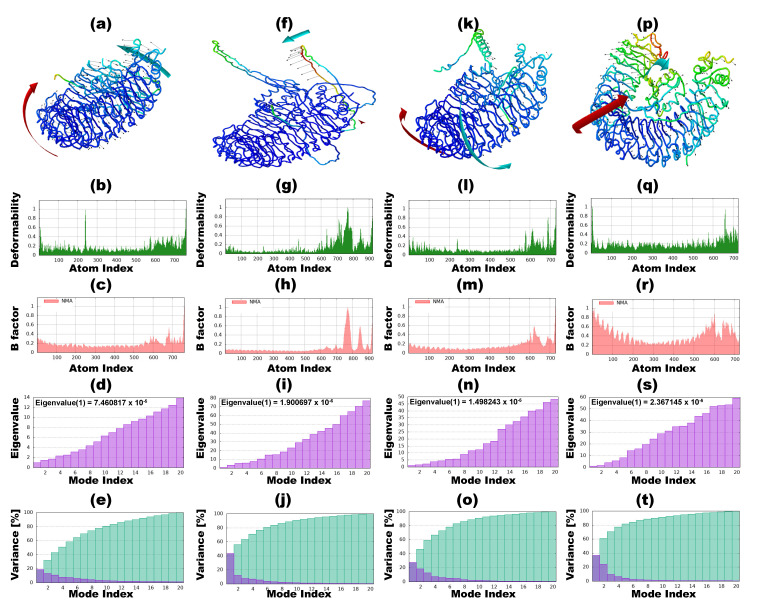
Normal mode analysis (NMA) of vaccine candidates against *T. rubrum* and TLR4 receptor complexes by iMODs. (**a**–**e**) iMODS results of BGTV-TLR24 complex. (**a**) NMA mobility; (**b**) main-chain deformability; (**c**) B-factor values; (**d**) the eigenvalue; (**e**) variance. (**f**–**j**) iMODS results of CDPV-TLR4 complex. (**f**) NMA mobility; (**g**) main-chain deformability; (**h**) B-factor values; (**i**) the eigenvalue; (**j**) variance; (**k**–**o**) iMODS results of GMPV-TLR4 complex. (**k**) NMA mobility; (**l**) main-chain deformability; (**m**) B-factor values; (**n**) the eigenvalue; (**o**) variance; (**p**–**t**) iMODS results of LDPV-TLR4 complex. (**p**) NMA mobility; (**q**) main-chain deformability; (**r**) B-factor values; (**s**) the eigenvalue; (**t**) variance.

The investigation involved simulations assessing the interactions of vaccine candidates against *T. rubrum* with immune receptors through NMA. The outcomes delineate BGTV, CDPV, GMPV, and LDPV docking complexes with TLR2 and TLR4 receptors, as shown in Figure 6 and Figure 7, respectively. Within these figures, panels (b, g, l, and q) spotlight the deformability graphs, emphasizing hinge points denoting notable deformability regions within the complexes. B-factor values were computed to quantify the uncertainties associated with the atomic positions within the docking complexes, providing a root mean square assessment. These values are visually depicted in Figure 6 and Figure 7, specifically in panels (c, h, m, and r). Eigenvalues pertaining to the BGTV-TLR2, CDPV-TLR2, GMPV-TLR2, LDPV-TLR2, BGTV-TLR4, CDPV-TLR4, GMPV-TLR4, and LDPV-TLR4 complexes were determined to be 1.38 × 10^−5^, 9.3 × 10^−7^, 2.9 × 10^−6^, 1.6 × 10^−5^, 7.4 × 10^−5^, 1.9 × 10^−6^, 1.5 × 10^−5^, and 2.36 × 10^−5^, respectively (as depicted in Figure 6d,i,n,s and Figure 7d,i,n,s). Supplementary Figures S4 and S5 augment the analysis by presenting the covariance matrices, delineating the associations between pairs of residues exhibiting correlated (red), uncorrelated (white), and anti-correlated (blue) motions. Furthermore, the elastic docking network (dark gray) depicted within these Supplementary Figures elucidates the relational dynamics between the atoms comprising the vaccine candidates and the TLR2/TLR4 receptor complexes.

### 3.10. Molecular Dynamic Simulation

**Figure 8:**
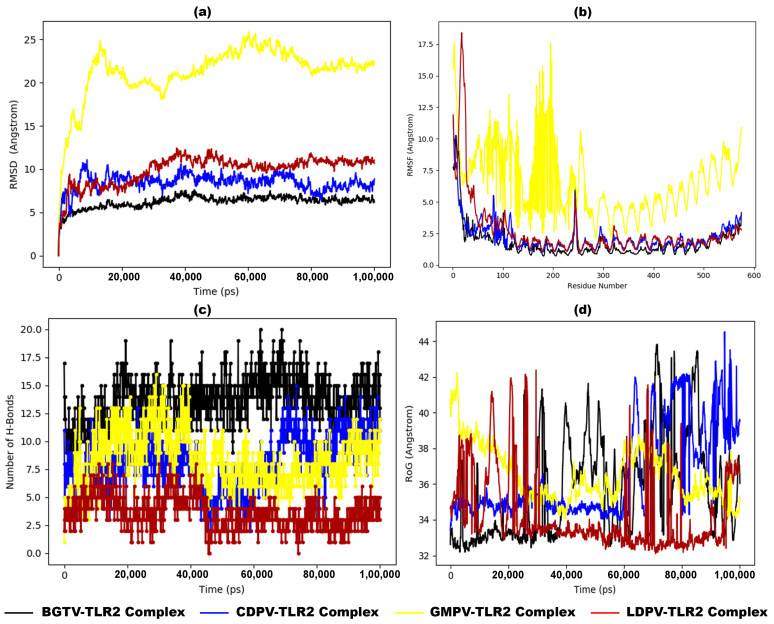
MD simulation results of dock complexes of potential vaccine candidates (BGTV (black), CDPV (blue), GMPV (yellow), and LDPV (red)) with TLR2 backbone. (**a**) Trajectory analysis of the RMSD between C-alpha atoms of dock complexes over time, (**b**) RMSF plot, (**c**) number of hydrogen bond formations, and (**d**) radius of gyration (RoG) plot.

**Figure 9:**
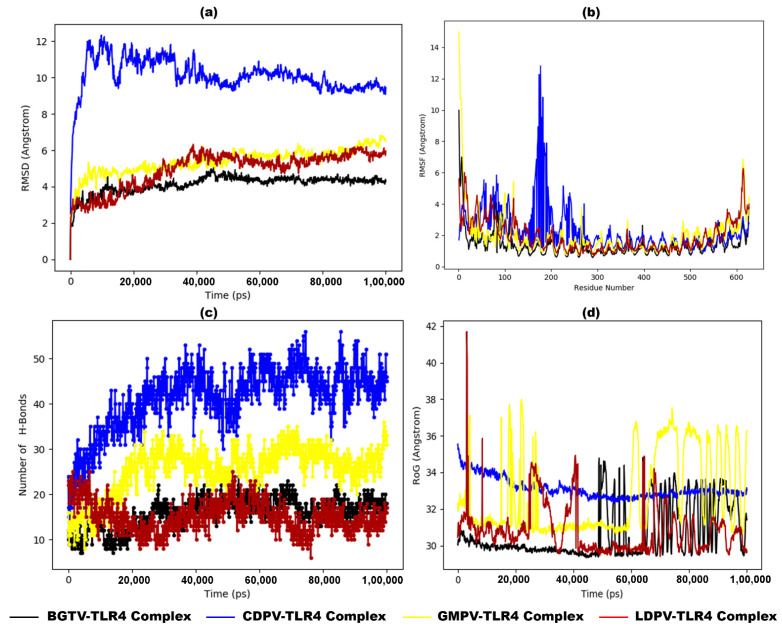
MD simulation results of dock complexes of potential vaccine candidates (BGTV (black), CDPV (blue), GMPV (yellow), and LDPV (red)) with TLR4 backbone. (**a**) Trajectory analysis of the RMSD between C-alpha atoms of dock complexes over time, (**b**) RMSF plot, (**c**) number of hydrogen bond formations, and (**d**) radius of gyration (RoG) plot.

MD simulation revealed the stability and dynamics of these vaccines and TLR complexes. Figure 8a presents the RMSD plot for the vaccine–TLR2 complexes, illustrating that the RMSD fluctuations for BGTV, CDPV, and LDPV with TLR2 are relatively stable, remaining below or near 0.9 nm. In contrast, the RMSD values for GMPV-TLR2 exhibit significant fluctuations after 15 ns, reaching up to 2.0 nm. Similarly, Figure 9a displays the RMSD plot for the vaccine–TLR4 complexes, indicating that the RMSD fluctuations for BGTV, CDPV, GMPV, and LDPV with TLR4 are stable, staying below 0.9 nm. Although CDPV-TLR4 initially shows high fluctuations around 1.0 nm at the beginning of the simulation, it stabilizes to 0.9 nm after 50 ns. These findings suggest that the docked vaccines exert a stabilizing effect on both TLR2 and TLR4 receptors.

The Root Mean Square Fluctuation (RMSF) analysis offers detailed insights into the regions of the studied proteins responsible for the observed RMSD fluctuations in the vaccine/TLR2 and vaccine/TLR4 systems. The RMSF values for the TLR4 backbone and the vaccine backbones show minimal fluctuations (Figure 9b). In contrast, the RMSF analysis of the TLR2 backbone indicates that GMPV is highly dynamic and exhibits significant fluctuations, while the other vaccines remain relatively conserved (Figure 8b). Notably, the RMSF profiles of vaccine–TLR2/TLR4 complexes closely resemble the fluctuation patterns observed in the NMA results from iMODS (as depicted in Figure 6c,h,m,r and Figure 7c,h,m,r). The radius of gyration, a critical parameter indicating structural compactness, revealed that the vaccine molecules attained a stable and compact form during the MD simulations (Figure 8d and Figure 9d). The compactness of the TLR2 and TLR4 complexes is attributed to the strong binding interactions of the designed vaccines. The stability of these interactions was evaluated by estimating the hydrogen bonding between the vaccines and TLR2 and TLR4 systems over 100 ns. The number of hydrogen bonds in the vaccine–TLR2 complexes remained constant over time (Figure 8c), while the number of hydrogen bonds in the vaccine–TLR4 complexes (Figure 9c) increased.

### 3.11. Immune Simulation Analysis

**Figure 10:**
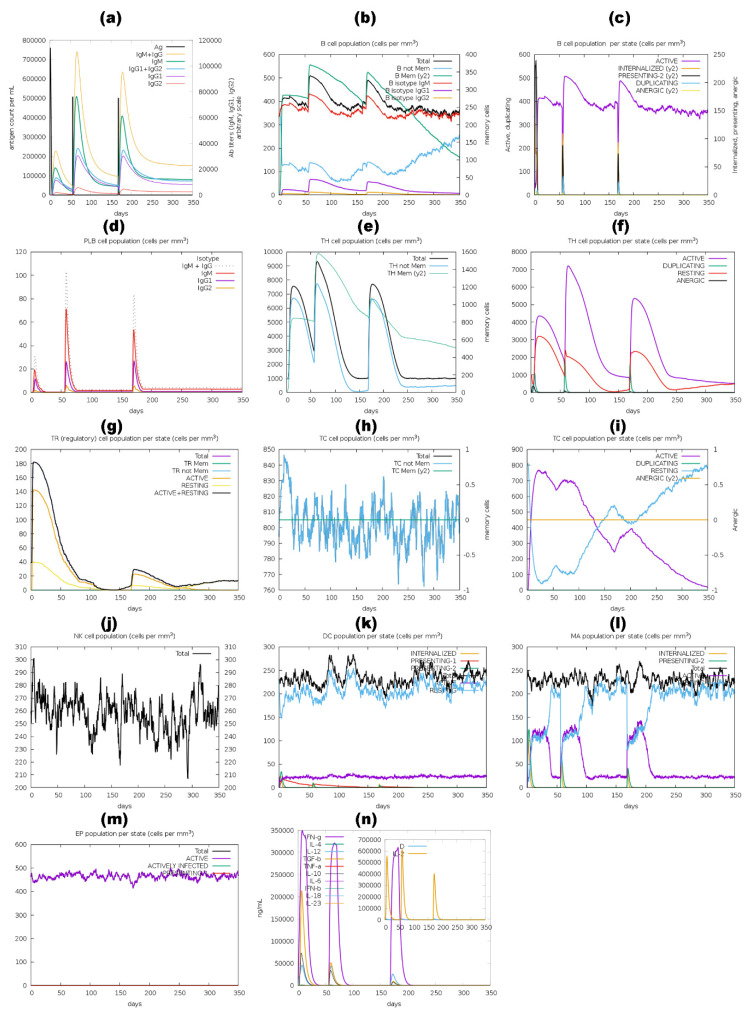
A computer-based simulation to model the immune response to the BGTV candidate, administering three doses over 350 days. Key parameters evaluated included antigen and immunoglobulins levels (**a**), LBLs (**b**–**d**), HTLs and CTLs (**e**–**i**), natural killer cells (**j**), dendritic cells (**k**), macrophages (**l**), epithelial presenting cell population (**m**), and cytokine concentrations (**n**). The Simpson index (D) was utilized to assess the simulation outcomes.

**Figure 11:**
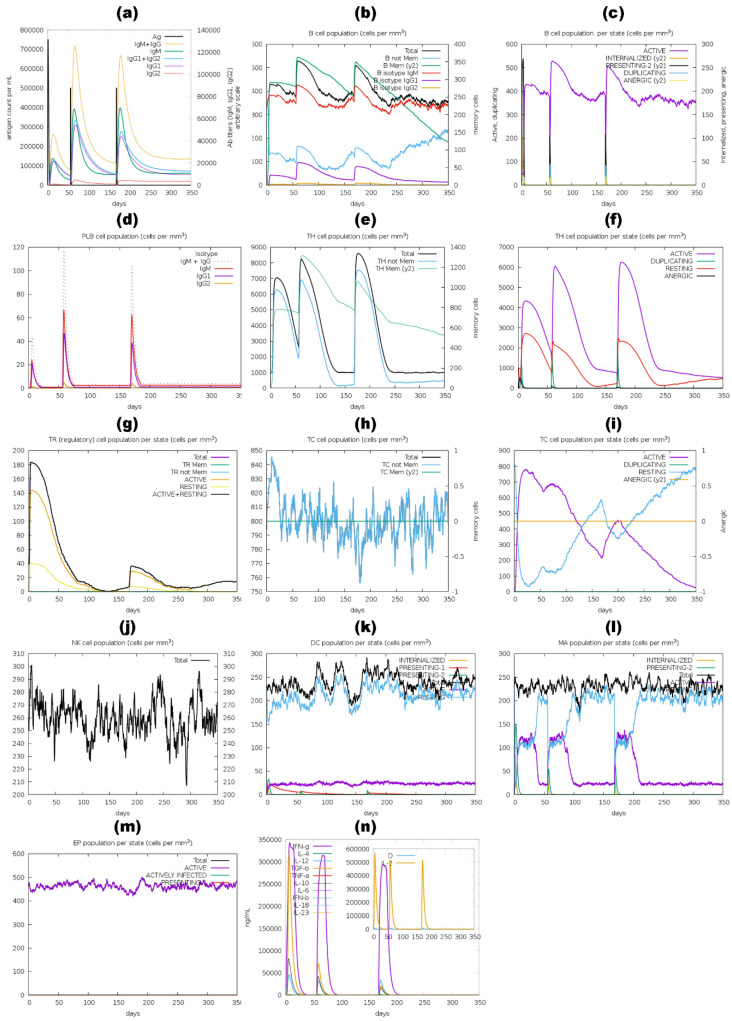
A computer-based simulation to model the immune response to the CDPV candidate, administering three doses over 350 days. Key parameters evaluated included antigen and immunoglobulins levels (a), LBLs (b–d), HTLs and CTLs (e–i), natural killer cells (j), dendritic cells (k), macrophages (l), epithelial presenting cell population (m), and cytokine concentrations (n). The Simpson index (D) was utilized to assess the simulation outcomes.

**Figure 12:**
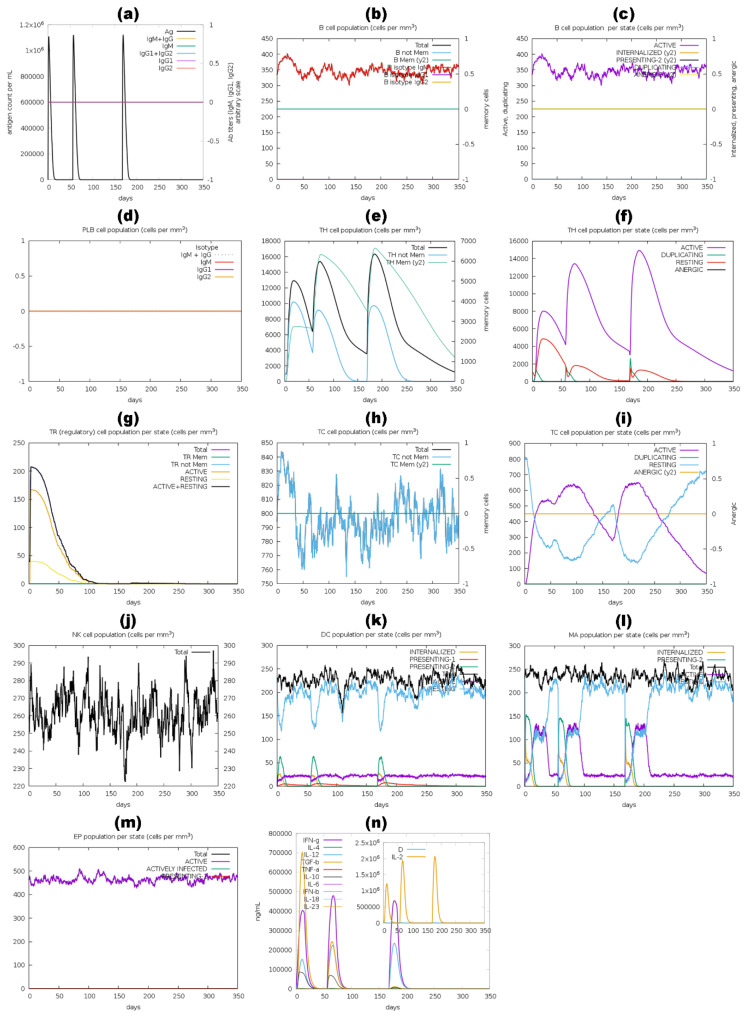
A computer-based simulation to model the immune response to the GMPV candidate, administering three doses over 350 days. Key parameters evaluated included antigen and immunoglobulins levels (a), LBLs (b–d), HTLs and CTLs (e–i), natural killer cells (j), dendritic cells (k), macrophages (l), epithelial presenting cell population (m), and cytokine concentrations (n). The Simpson index (D) was utilized to assess the simulation outcomes.

**Figure 13:**
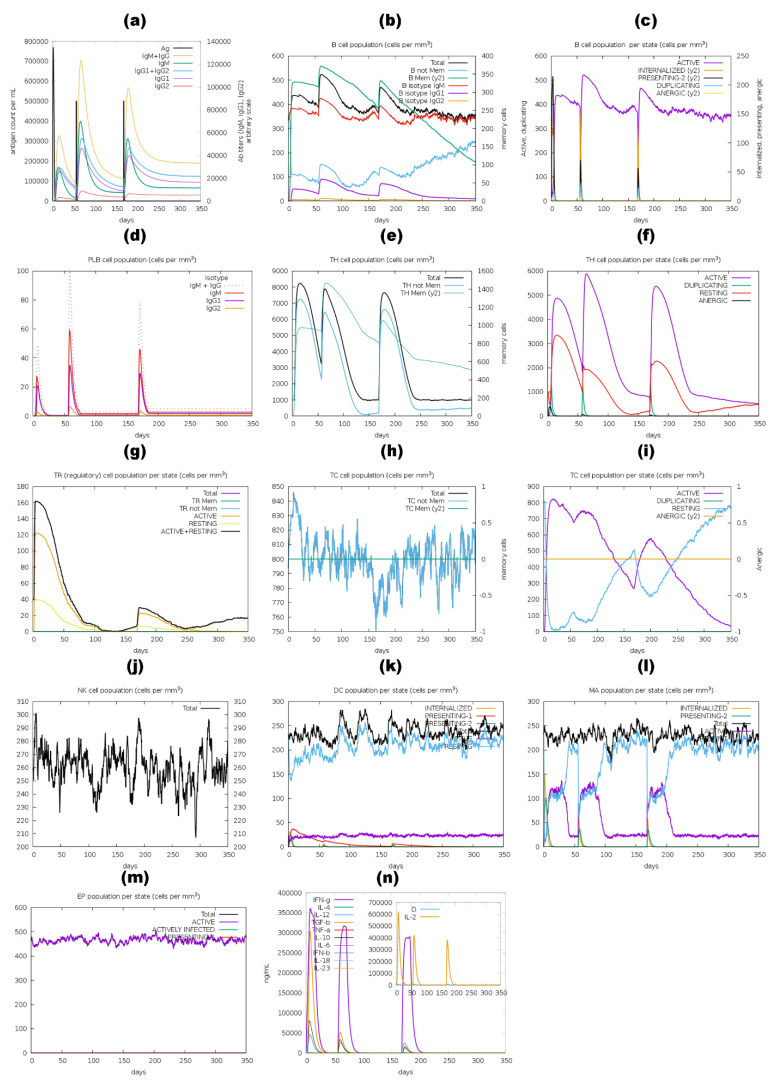
A computer-based simulation to model the immune response to the LDPV candidate, administering three doses over 350 days. Key parameters evaluated included antigen and immunoglobulins levels (a), LBLs (b–d), HTLs and CTLs (e–i), natural killer cells (j), dendritic cells (k), macrophages (l), epithelial presenting cell population (m), and cytokine concentrations (n). The Simpson index (D) was utilized to assess the simulation outcomes.

The immune stimulation by the C-ImmSim server showed results consistent with actual immune responses. Increased levels of IgM, as shown in Figure 10a,d, Figure 11a,d, Figure 12a,d and a,d, characterized the primary reaction after the first BGTV, CDPV, and LDPV dose. After the second and third BGTV, CDPV, and LDPV doses, there was a remarkable increase in IgG1, IgG1  +  IgG2, IgM, and IgG  +  IgM antibody levels. The IgM  +  IgG levels peaked after the third dose of BGTV, CDPV, and LDPV, reaching over 200,000, indicating a robust humoral immune response, as evidenced by the subsequent decrease in antigen population (Figure 10a,d, Figure 11a,d, and Figure 13a,d). In contrast, after inoculation of three doses of GMPV, no immunogenic response was observed (as shown in Figure 12a,d). 

Similarly, the active B cell populations remained elevated for up to 150 cells per mm^3^ after three BGTV, CDPV, and LDPV doses, as shown in Figure 10b,c, Figure 11a,d, and Figure 13b,c. For the GMPV, no active B cells were observed in all three doses (Figure 12b,c). The total active HTL was sustainably elevated after inoculating the BGTV, CDPV, GMPV, and LDPV (Figure 10e,f, Figure 11e,f, and Figure 13e,f). The resting active regulatory HTL concentrations were high after the first dose of BGTV, CDPV, GMPV, and LDPV and gradually decreased over time (Figure 10g, Figure 11g, Figure 12g and Figure 13g). The cytotoxic HTL concentration also varied for BGTV, CDPV, GMPV, and LDPV (Figure 10h, Figure 11h, Figure 12h and Figure 13h). Their active form constantly decreased with vaccination (Figure 10i, Figure 11i, Figure 12i and Figure 13i). The natural killer cell (NKC) population also varied (Figure 10j, Figure 11j, Figure 12j and Figure 13j), as the numbers of dendritic cells, macrophages, and epithelial presenting cells were constant in cells per mm^3^, as shown in Figure 10k–m, Figure 11k–m, Figure 12k–m and Figure 13k–m. After the injection of vaccine candidates, different cell activations elevated the immune response, activating cytokines and interleukins, primarily IFN-γ and IL-2 (Figure 10n, Figure 11n, Figure 12n and Figure 13n).

In parallel, immunoglobulin levels of BGTV, CDPV, GMPV, and LDPV without adjuvants were evaluated, as shown in Supplementary Figure S6. Interestingly, the immunoglobulin production patterns of all the vaccines without adjuvants were similar to those with adjuvants; for instance, the primary reaction after the first dose showed increased levels of IgM. After the second and third doses, there was a significant rise in IgG1, IgG1  +  IgG2, IgM, and IgG  +  IgM antibodies. IgM  +  IgG levels peaked after the third dose of BGTV, CDPV, and LDPV without adjuvant, surpassing 200,000, indicating a strong humoral immune response, which led to a decrease in the antigen population. In contrast, three doses of GMPV without adjuvant did not induce any immunogenic response.

## 4. Discussion

Recent advances in bioinformatics, structural biology, and computational tools have revolutionized vaccine development [133]. In silico studies for predicting and designing vaccines have expanded significantly, encompassing bacteria, viruses, fungi, and even cancer [134]. The ongoing COVID-19 pandemic underscores the urgent need for effective strategies to manage opportunistic infections and safeguard immunocompromised individuals [135]. Developing an efficacious vaccine against tinea cruris has become a paramount health priority.

Applying in silico approaches in vaccine design and validation offers considerable time and cost savings. Computational tools expedite proteome analysis and identification of potential vaccine candidates, thus proving particularly beneficial for infections challenging to cultivate or caused by diverse infectious agents, like *T. rubrum*. Mapping epitopes and conducting docking analyses with corresponding receptors provide valuable insights into epitope behavior upon encountering human immune receptors. As a result, computational tools offer initial validation before costly laboratory experiments, representing a cost-effective approach. Given these advantages, reverse vaccinology and immunoinformatics approaches centered on computational vaccine design and analysis have gained traction in recent years. These methods have demonstrated promising results in various studies, offering practical validation of computational prediction methods when coupled with wet-lab experiments on designed vaccines [136,137,138,139,140].

Treating fungal infections, notably tinea cruris, poses several hurdles. Recalcitrant infections and fungal resistance are significant challenges, often exacerbated by self-treatment with over-the-counter topical antifungal and steroid preparations [141,142]. Emerging formulations like luliconazole and overlooked agents like ciclopirox offer potential solutions against fungal resistance [143]. However, the use of topical steroids for tinea cruris remains contentious due to conflicting data and ongoing investigations into their efficacy [3]. Moreover, determining the appropriate dose and duration of systemic therapies for tinea cruris and tinea pedis remains uncertain, highlighting the need for well-designed trials and evidence-based guidelines. The shifting epidemiology of dermatophytosis, particularly its rising prevalence in tropical and subtropical regions like India, raises concerns. Urbanization and factors like occlusive footwear and tight clothing contribute to this trend [9].

The pathogenesis of dermatophytosis often involves genetic predispositions, potentially influenced by specific defects in innate and adaptive immunity [144]. Topical antifungals such as butenafine and terbinafine have demonstrated superiority over clotrimazole, with terbinafine outperforming ciclopirox and naftifine surpassing oxiconazole [9]. Proper diagnosis, appropriate selection of antifungal agents, adherence to recommended therapy duration, avoidance of steroids, patient education on lifestyle modifications, and responsible use of antifungal medications are potential strategies to address current challenges [3,9,141,144]. Efforts to combat fungal infections through vaccine development are advancing, driven by the rising prevalence and resistance to antifungal drugs. The current study investigated the whole proteome of *T. rubrum* (the leading cause of tinea cruris) to recruit potential protein candidates for vaccine targets. For this reason, we recruited four proteins (1,3-beta-glucanosyltransferase, CFEM domain-containing protein, cell wall galactomannoprotein, and LysM domain-containing protein) that not only meet the criteria of vaccine targets (Table 1) but also play pivotal roles in cell wall biosynthesis, pathogenesis, virulence, and interactions with the host immune system in *T. rubrum*. 

1,3-beta-glucanosyltransferase involves elongating and branching beta-(1,3)-glucan, a fundamental constituent of the fungal cell wall meshwork. Its activity is indispensable for the fungal cell wall’s structural integrity, complexity, and functionality [145,146,147]. CFEM domain-containing proteins, characterized by eight conserved cysteine residues, are integral to numerous fungal functions, encompassing iron acquisition, cell wall stability, and pathogenesis. These proteins mediate interactions with host organisms, influencing fungal survival strategies and virulence [148,149,150,151,152]. 

Galactomannoproteins are essential constituents of the fungal cell wall, contributing significantly to its structural integrity and functional properties. Their involvement spans fungal pathogenesis, virulence, and host immune system interactions. Notably, their absence in humans makes them promising targets for antifungal therapies [55,153,154]. LysM domain-containing proteins play pivotal roles in fungal biology, particularly in interactions with the environment and host organisms. These proteins are implicated in pathogenesis, virulence, fungal growth, morphogenesis, spore germination, and immune evasion. Through binding to chitin, a major component of fungal cell walls, LysM proteins influence fungus–host interactions, potentially altering host physiology to facilitate fungal colonization and pathogenicity [155,156,157,158,159].

The physicochemical properties of four recruited proteins (Table 2) exploited valuable knowledge about protein characteristics, contributing significantly to diverse scientific domains, including pharmaceutical research, personalized medicine, and biomarker discovery. For instance, identifying the optimal pI value of a protein intended for vaccination can enhance its ability to trigger a robust immune response. Furthermore, it can ensure the protein’s stability, solubility, and effective interaction with the immune system, augmenting its capacity to elicit protective immune responses [160,161,162]. The sorted sequences of the CFEM domain-containing protein and cell wall galactomannoprotein were unstable. However, these proteins’ avirulence factors and other functions, which may not necessarily imply instability, are noteworthy. For instance, fungi’s cell wall/membrane CFEM domains are unique and closely related to pathogenicity. Therefore, the stability of these proteins in *T. rubrum* cannot be directly inferred. [150,163].

Further, the computational antigenic, immunogenic, non-toxic, and non-allergenic epitope profiling explored the candidates for vaccine construction (as shown in Table 3, Table 4 and Table 5). The epitopes were linked via linkers that are pivotal in designing multi-epitope vaccine constructs, contributing to their structural integrity and immunogenicity. For example, the AAY linker prevents domain disruption within the vaccine construct. The GPGPG linker, comprising flexible and hydrophilic amino acids, is instrumental in maintaining structural integrity. The KK linker facilitates the effective connection of linear B-cell epitopes. Additionally, the EAAAK linker enhances the immunogenic properties of the vaccine by leveraging its rigidity and propensity for helix formation [87,164,165,166,167]. 

Including Toll-like receptor (TLR) ligands, such as RS09, as adjuvants in human vaccines has demonstrated promising results in augmenting protection against infectious diseases. TLR-based adjuvants, like Pam3CysSerLys4 (Pam3CSK4), have been identified as potent stimulators of B-cell responses and T-cell activation, thereby enhancing vaccine efficacy [168]. Incorporating RS09 and other TLR agonists into vaccine formulations represents a significant advancement in vaccine development, aiming to elicit robust and targeted immune responses against pathogens, including fungi and viruses [168,169,170]. Research has explored the TLR4 agonist RS09 as an adjuvant in fungus vaccine development [88,89,90]. RS09, which mimics lipopolysaccharide (LPS), a natural TLR4 ligand, has been incorporated into vaccine constructs to enhance immune responses. In designing a multivalent vaccine against *Candida albicans*, RS09 was strategically positioned at the N-terminal end of the final vaccine construct to facilitate co-stimulation of T-cell receptors (TCRs), leading to heightened immune activation [88]. This synthetic adjuvant, RS09, offers a safer alternative to traditional adjuvants like Freund’s adjuvant, with its presence in the vaccine construct aimed at effectively stimulating TCRs to drive a robust immune response.

The protein-based vaccines underwent reverse translation to construct the mRNA vaccines along with 5′ m7GCap, 5′ UTR, Kozak sequence, tPA signal peptide, and EAAAK linker at the N-terminal and MITD sequence, stop codon, 3′ UTR, and poly (A) tail at the C-terminal (as shown in Supplementary Table S1). The mRNA vaccine is first delivered into immune cells, such as dendritic cells and macrophages [101]. Once inside the immune cells, the mRNA escapes the endosomes and releases into the cytosol. The host cell’s ribosomes translate the mRNA into the desired antigenic protein. This protein is subsequently degraded in the proteasomes and presented to the immune system via MHC class I and II pathways, triggering an adaptive immune response. Additionally, the mRNA vaccine stimulates the innate immune response by activating pattern recognition receptors (PRRs) and releasing pro-inflammatory cytokines, further enhancing the immune response. This immune activation produces antibodies that recognize and mark the pathogen for destruction, protecting against future infections. The vaccine also induces cellular immunity by activating CD8+ and CD4+ T cells, which help eliminate infected cells and provide long-term immunity. These steps collectively enable mRNA vaccines to elicit a robust immune response against specific pathogens, making them a promising alternative to traditional vaccine approaches [49,101]. 

The docking and NMA analysis of vaccine candidates with TLRs confirmed a significant binding affinity and structural deformability, showing the stability of complexes and the vaccines’ promising ability to activate the immune response. The immune simulation analysis of vaccines with and without adjuvants confirmed the claim of immune response activation by showing elevated results of IgG1, IgG1  +  IgG2, IgM, and IgG  +  IgM antibodies and immune cells activating cytokines and interleukins. No significant change was observed in antibody production between vaccines with and without adjuvants. However, the adjuvant plays a crucial role in activating the innate immune response by interacting with TLRs [88,89,90].

Currently, no mRNA vaccines have been approved to prevent or treat fungal infections. However, research is ongoing to explore the potential of mRNA vaccines in this area. For example, a recent study investigated the use of mRNA vaccines to protect against the fungal pathogen *Candida albicans*. The study found that mRNA vaccines encoding specific fungal antigens could induce a protective immune response in mice, suggesting that mRNA vaccines could be a promising approach for preventing and treating fungal infections [21]. In the context of fungal infections, mRNA vaccines could stimulate the immune system to produce antibodies against specific fungal antigens, potentially leading to protection against infection. However, more research is needed to determine the safety and effectiveness of mRNA vaccines against fungal infections. Although the immunoinformatic approach holds promise, the lack of a standardized benchmark for fungal vaccine development and limited understanding of their pathogenesis and adaptive immune system response may hinder its efficacy. As a result, experimental validation, encompassing both in vivo and in vitro studies, is essential to assess the immunogenicity, effectiveness, and safety of newly developed vaccines.

## 5. Conclusions

Based on the immunoinformatic analyses, our designed mRNA-based fungal vaccines exhibit promising potential to induce efficient immunogenicity against *T. rubrum* fungal infections. Scalable and cost-effective production and comprehensive product characterization can address the increasing need for mRNA vaccines targeting various infectious diseases, particularly challenging fungal infections, which have thick fungal cell walls, similarities between fungal and human cells, antigenic variation, and evolutionary resemblance to animals. The proposed mRNA vaccine constructs meet criteria for antigenicity, immunogenicity, allergenicity, toxicity, and other physicochemical properties, indicating stability and safety, potentially offering long-term immunity and reducing reliance on antifungal medications. Nevertheless, further preclinical studies and validation are imperative before initiating both in vivo and in vitro experimental clinical trials to validate the study findings.

## Figures and Tables

**Table 1:** Proteins extracted from *Trichophyton rubrum* proteomic dataset meeting criteria of targeted for vaccine design.

Protein ID	Protein Name	Length	Antigenicity	Localizations	Extra Cellularity Score	Human Homolog Identity	Transmembrane Helix Score	Molecular Weight
F2SF86	1,3-beta-glucanosyltransferase	531	0.74	Extracellular	0.8228	0.00%	0	57.45 kDa
F2SCX9	CFEM domain-containing protein	263	1.13	Extracellular	0.8623	0.00%	0	24.95 kDa
F2SDA6	Cell wall galactomannoprotein	177	0.67	Extracellular	0.9471	0.00%	0	18.99 kDa
A0A080WV70	LysM domain-containing protein	283	0.98	Extracellular	0.942	0.00%	0	31.15 kDa

**Table 2:** Physiochemical properties of sorted proteins from *T. rubrum* targeted for vaccine design.

Physiochemical Properties	1,3-Beta-glucanosyltransferase	CFEM Domain-Containing Protein	Cell Wall Galactomannoprotein	LysM Domain-Containing Protein
**Number of amino acids**	531	263	177	283
**Theoretical pI (ExPASy-ProtParam)**	5.73	4.66	5.51	6.57
**Theoretical pI (EMBOSS-PEPSTATS)**	5.63	4.37	5.32	6.96
**Negatively charged residues (Asp + Glu)**	57	18	23	18
**Positively charged residues (Arg + Lys)**	54	7	21	17
**Formula**	C_2531_H_3924_N_658_O_807_S_30_	C_1029_H_1666_N_298_O_397_S_12_	C_839_H_1363_N_225_O_258_S_8_	C_1375_H_2135_N_365_O_418_S_21_
**Total number of atoms**	7950	3402	2693	4314
**Ext. coefficient (ExPASy-ProtParam)**	64,595	3480	3105	39,015
**Molar ext. coefficients (EMBOSS-PEPSTATS)**	63,720 (reduced), 64,595 (cystine bridges)	2980 (reduced), 3480 (cystine bridges)	2980 (reduced), 3105 (cystine bridges)	38,390 (reduced), 39,015 (cystine bridges)
**Estimated Half-life (mammalian reticulocytes, in vitro) (hours)**	>30	>30	>30	>30
**Estimated Half-life (yeast, in vivo) (hours)**	>20	>20	>20	>20
**Estimated Half-life (Escherichia coli, in vivo) (hours)**	>10	>10	>10	>10
**Instability index**	25.42	47.14	50.6	26.67
**Stability classification**	Stable	Unstable	Unstable	Stable
**Aliphatic index**	65.59	53.12	90.51	72.76
**Grand average of hydropathicity (GRAVY)**	−0.333	−0.244	0.013	−0.142
**Solubility**	0.475	0.148	0.306	0.271
**Improbability of expression in inclusion bodies**	0.816	0.97	0.571	0.773

**Table 3:** NetMHCpan EL 4.1 method on the IEDB server predicted antigenic CTL binding epitopes of sorted proteins from *T. rubrum* targeted for vaccine design.

Proteins	Position	Peptide	Antigenic Score	Toxin	Immunogenicity Score	Allergen
**1,3-beta-glucanosyltransferase**	36–44	SNGTEFFMK	0.78	No	0.25	No
	63–71	SYQDPLADV	1.5	No	0.01	No
	82–90	QELQTNTIR	0.94	No	0.02	No
	83–91	ELQTNTIRV	1.99	No	0.18	No
	137–145	YTRYTSVID	1.37	No	0.007	No
	150–158	YTNVIGFFA	1.4	No	0.37	No
	151–159	TNVIGFFAG	1.8	No	0.41	No
	194–202	FWGYNIYSW	2.2	No	0.02	No
	222–230	NFNVPVFFA	1.08	No	0.23	No
**CFEM domain-containing protein**	22–30	THVVTTSRP	1.08	No	0.02	No
	27–35	TSRPPTTLY	0.83	No	0.05	No
	28–36	SRPPTTLYT	1.75	No	0.04	No
	33–41	TLYTEVSGS	1.4	No	0.05	No
	47–55	SSSPTGTGS	1.64	No	0.05	No
	48–56	SSPTGTGSE	1.96	No	0.03	No
	49–57	SPTGTGSES	1.17	No	0.03	No
	66–74	PSSTEGGSS	1.7	No	0.04	No
**Cell wall galactomannoprotein**	50–58	AQSPGGITE	1.98	No	0.13	No
	60–68	MSVTNDIYD	0.59	No	0.17	No
**LysM domain-containing protein**	73–81	PSTTTTAKP	0.67	No	0.03	No
	101–109	TRAMTTTIS	2.33	No	0.02	No

**Table 4:** Antigenic HTL binding epitopes of sorted proteins from *T. rubrum* targeted for vaccine design, predicted using IEDB recommended 2.22 method on the IEDB server.

Proteins	Position	Peptide	Antigenic Score	Toxin	IFN	IL4 Inducer	IL10 Inducer	Allergen
**1,3-beta-glucanosyltransferase**	58–72	TSADNSYQDPLADVK	0.7	No	Positive	Yes	Yes	No
	59–73	SADNSYQDPLADVKS	0.91	No	Positive	Yes	Yes	No
**CFEM domain-containing protein**	24–38	VVTTSRPPTTLYTEV	0.7	No	Positive	No	No	No
	25–39	VTTSRPPTTLYTEVS	1.07	No	Positive	No	No	No
	27–41	TSRPPTTLYTEVSGS	1.28	No	Positive	No	No	No
	29–43	RPPTTLYTEVSGSQT	1.71	No	Positive	No	No	No
	88–102	TSGSGNGPSQTPSQG	1.0	No	Positive	No	No	No
	89–103	SGSGNGPSQTPSQGI	1.03	No	Positive	No	No	No
	90–104	GSGNGPSQTPSQGIA	1.03	No	Positive	No	No	No
	91–105	SGNGPSQTPSQGIAP	0.57	No	Positive	No	No	No
**Cell wall galactomannoprotein**	45–59	LSQRIAQSPGGITEL	1.87	No	Positive	No	No	No
	111–125	ATSTKVPLIKAVPGG	1.7	No	Positive	No	No	No
**LysM domain-containing protein**	99–113	TTTRAMTTTISSDAP	1.55	No	Positive	Yes	No	No
	138–152	SIQTKYGISTDQFKA	2.43	No	Positive	Yes	No	No
	139–153	IQTKYGISTDQFKAW	2.58	No	Positive	Yes	No	No
	141–155	TKYGISTDQFKAWNP	1.94	No	Positive	Yes	No	No
	142–156	KYGISTDQFKAWNPY	1.99	No	Positive	Yes	No	No
	146–160	STDQFKAWNPYINAE	1.81	No	Positive	Yes	No	No
	143–157	YGISTDQFKAWNPYI	2.3	No	Positive	Yes	No	No

**Table 5:** Predicted sorted protein LBL epitopes of *T. rubrum* using IEDB’s BepiPred Linear Epitope Prediction 2.0 methods.

Proteins	Position	Peptide	Length	Antigenicity Score	Toxin	Allergen
**1,3-beta-glucanosyltransferase**	235–253	NEVQPRMFTEVQALYGDKM	19	0.7053	No	No
**CFEM domain-containing protein**	53–251	CSNADFQHGLRDCTHEACPGEKVEQVVQAGLQACREMGGAPGSSTGAPTTGTGSGTTTGTPTSGSGSETTAPSTSGSGSAPAPTSGGHSTPYSTIPAGPTVITSGTHVVTTSRPPTTLYTEVSGSQTGSESSSPTGTGSESTSAPETTSPSSTEGGSSPSSTEGSGNGGSGGSETSGSGNGPSQTPSQGIAPKATGLGV	199	1.152	No	No
**Cell wall galactomannoprotein**	22–61	PSTFSSVPEAIGDLDPISASIEGLSQRIAQSPGGITELMS	40	1.297	No	No
**LysM domain-containing protein**	176–202	GATISTSMPMPTPSGPQPQMPGIVSNC	27	0.5542	No	No

**Table 6:** Protein-based vaccine sequences targeting 1,3-beta-glucanosyltransferase, CFEM domain-containing protein, cell wall galactomannoprotein, and LysM domain-containing protein with their profiling (adjuvant: red, linkers: green, cyan, blue, and mustard, B-cell epitope: pink, HTL: yellow, CTL: grey, and His tag: purple).

Vaccines	BGTV	CDPV	GMPV	LDPV
**Targeting Proteins**	1,3-beta-glucanosyltransferase	CFEM domain-containing protein	Cell wall galactomannoprotein	LysM domain-containing protein
**Vaccine Sequence**	APPHALS EAAAK NEVQPRMFTEVQALYGDKM KK TSADNSYQDPLADVKS AAY SNGTEFFMK GPGPG SYQDPLADV GPGPG QELQTNTIRV GPGPG YTRYTSVID GPGPG YTNVIGFFAG GPGPG FWGYNIYSW GPGPG NFNVPVFFA GPGPG HHHHHH	APPHALS EAAAK CSNADFQHGLRDCTHEACPGEKVEQVVQAGLQACREMGGAPGSSTGAPTTGTGSGTTTGTPTSGSGSETTAPSTSGSGSAPAPTSGGHSTPYSTIPAGPTVITSGTHVVTTSRPPTTLYTEVSGSQTGSESSSPTGTGSESTSAPETTSPSSTEGGSSPSSTEGSGNGGSGGSETSGSGNGPSQTPSQGIAPKATGLGV KK VVTTSRPPTTLYTEVSGSQT AAY TSGSGNGPSQTPSQGGIAP AAY THVVTTSRPPTTLYTEVSGS GPGPG SSSPTGTGSES GPGPG PSSTEGGSS GPGPG HHHHHH	APPHALS EAAAK PSTFSSVPEAIGDLDPISASIEGLSQRIAQSPGGITELMS KK LSQRIAQSPGGITEL AAY ATSTKVPLIKAVPGG AAY AQSPGGITE GPGPG MSVTNDIYD GPGPG HHHHHH	APPHALS EAAAK GATISTSMPMPTPSGPQPQMPGIVSNC KK TTTRAMTTTISSDAP AAY SIQTKYGISTDQFKAWNPYINAE AAY PSTTTTAKP GPGPG TRAMTTTIS GPGPG HHHHHH
**Number of Amino Acids**	158	319	124	119
**Molecular Weight (Da)**	16,673.4	30,329.02	12,476.94	12,292.77
**Theoretical pI (ExPASy-ProtParam)**	6.14	5.68	6.14	9.47
**Theoretical pI (EMBOSS-PEPSTATS)**	6.6	6.05	6.6	9.8
**Negatively Charged Residues (Asp + Glu)**	12	19	10	4
**Positively Charged Residues (Arg + Lys)**	9	10	7	8
**Ext. coefficient (ExPASy-ProtParam)**	24,410	9190	4470	11,460
**Molar ext. coefficients (EMBOSS-PEPSTATS)**	24,410 (reduced), 24,410 (cystine bridges)	8940 (reduced), 9190 (cystine bridges)	4470 (reduced), 4470 (cystine bridges)	11,460 (reduced), 11,460 (cystine bridges)
**Estimated Half-life (mammalian reticulocytes, in vitro)**	4.4 h	4.4 h	4.4 h	4.4 h
**Estimated Half-life (yeast, in vivo)**	>20 h	>20 h	>20 h	>20 h
**Estimated Half-life (*E. coli*, in vivo)**	>10 h	>10 h	>10 h	>10 h
**Instability Index (II)**	15.64	50.49	66.81	31.74
**Stability**	Stable	Unstable	Unstable	Stable
**Aliphatic Index**	46.96	33.39	75.73	42.1
**GRAVY**	−0.564	−0.653	−0.257	−0.571
**Antigenicity Score (VaxiJen 2.0)**	0.52	1.0026	0.9305	1.0933
**Antigenicity Score (ANTIGENpro)**	0.83	0.88	0.86	0.87
**Allergen Status**	Probable Non-Allergen	Probable Non-Allergen	Probable Non-Allergen	Probable Non-Allergen
**Toxin Status**	Non-Toxin	Non-Toxin	Non-Toxin	Non-Toxin
**Solubility**	0.563	0.520	0.435	0.294
**Improbability of expression in inclusion bodies**	0.964	0.965	0.92	0.978

**Table 7:** Comparative representation of Ramachandran plot statistics of unrefined and refined 3D constructs of BGTV, CDPV, GMPV, and LDPV.

Ramachandran Plot	BGTV		CDPV		GMPV		LDPV	
	Unrefined	Refined	Unrefined	Refined	Unrefined	Refined	Unrefined	Refined
**Residues in most favored regions**	60.90%	95.50%	30.80%	92.40%	57.60%	97.80%	42.90%	91.20%
**Residues in additional allowed regions**	23.60%	4.50%	31.20%	4.90%	17.40%	2.20%	41.80%	6.60%
**Residues in generously allowed regions**	13.60%	0.00%	25.00%	2.20%	15.20%	0.00%	11.00%	2.20%
**Residues in disallowed regions**	1.80%	0.00%	12.90%	0.40%	9.80%	0.00%	4.40%	0.00%

**Table 8:** Characteristic features of BGTV, CDPV, GMPV, and LDPV candidate docking complexes against *T. rubrum* and immune receptors (TLR2 and TLR4).

Docking Complex	Interface Residues	Interface Area (Å^2^)	dG (kcal mol^−1^)	Kd (M) at 25 °C	Binding Affinity in kcalmol^−1^ (Center, Lower Energy)	Electrostatic-Favored Binding Affinity in kcal mol^−1^ (Center, Lower Energy)	Hydrophobic-Favored Binding Affinity in kcal mol^−1^ (Center, Lower Energy)	Van-der Waal and Electrostatic Binding Affinity in kcal mol^−1^ (Center, Lower Energy)	Salt Bridges	Hydrogen Bonds	Non-Bonded Contacts
**BGTV-TLR2**	28–17	1056–1187	−13.5	1.2 × 10^−10^	−1077.2, −1124.9	−1084.7, −1134.4	−1857.4, −2124.7	−191.5, −264.3	2	12	150
**CDPV-TLR2**	29–10	785–1154	−14.5	2.2 × 10^−11^	−1059.4, −1114.9	−1003.9, −1100.0	−1645.9, −1783.9	−201.9, −247.2		4	146
**GMPV-TLR2**	31–13	864–1238	−12.7	5.2 × 10^−10^	−1062.7, −1205.8	−1184.4, −1271.5	−1883.9, −1962.8	−214.3, −253.6		6	153
**LDPV-TLR2**	39–27	1376–1709	−15.1	8.6 × 10^−12^	−1233.6, −1453.9	−1231.6, −1546.6	−1926.6, −2129.6	−242.2, −288.3	3	17	215
**BGTV-TLR4**	30–22	1205–1418	−13.5	1.4 × 10^−10^	−902.6, −993.5	−987.4, −1030.1	−941.4, −1189.2	−212.6, −212.6	6	14	150
**CDPV-TLR4**	51–51	2264–2314	−16.4	8.9 × 10^−13^	−916.9, −959.9	−883.9, −977.3	−988.9, −1092.8	−188.6, −219.4	3	30	317
**GMPV-TLR4**	39–33	1670–1791	−14.8	1.4 × 10^−11^	−911.4, −911.4	−817.3, −1016.6	−1062.7, −1174	−208.9, −213.5	4	17	226
**LDPV-TLR4**	49–38	1932–2137	−20.4	1.1 × 10^−15^	−1109.5, −1234.4	−1050.2, −1324.2	−1205.8, −1390.1	−210.1, −235.8	2	29	229

## Data Availability

The data presented in this study are available upon request from the corresponding author.

## Supplementary Materials

The following supporting information can be downloaded at: https://www.mdpi.com/article/10.3390/pharmaceutics16080983/s1, Figure S1: Revealing the minimal free energy (MFE) and stability of optimal secondary structures of mRNA BGTV (MFE (a) and Centroid (b)), CDPV (MFE (c) and Centroid (d)), GMPV (MFE (e) and Centroid (f)), and LDPV (MFE (g) and Centroid (h)) candidates; Figure S2: Ramachandran plots of 3D modeled structures of vaccines, i.e., BGTV (a), CDPV (b), GMPV (c), and LDPV (d); Figure S3: Spatial Distribution of Continuous and Discontinuous B-cell Epitopes in BGTV, CDPV, GMPV, and LDPV. Continuous (a) and Discontinuous (b) B-cell Epitopes of BGTV are shown in different color surfaces. Continuous (c) and Discontinuous (d) B-cell Epitopes of CDPV are shown in different color surfaces. Continuous (e) and Discontinuous (f) B-cell Epitopes of GMPV are shown in different color surfaces. Continuous (g) and Discontinuous (h) B-cell Epitopes of LDPV are shown in different color surfaces; Figure S4: Covariance and elastic network maps of vaccine candidates against *T. rubrum* and TLR2 receptor complexes by iMODs. (a) Covariance map and (b) elastic network of BGTV- TLR2; (c) Covariance map and (d) elastic network of CDPV-TLR2; (e) Covariance map and (f) elastic network of GMPV-TLR2; (g) Covariance map and (h) elastic network of LDPV- TLR2 docked complex; Figure S5: Covariance and elastic network maps of vaccine candidates against *T. rubrum* and TLR4 receptor complexes by iMODs. (a) Covariance map and (b) elastic network of BGTV- TLR4; (c) Covariance map and (d) elastic network of CDPV-TLR4; (e) Covariance map and (f) elastic network of GMPV-TLR4; (g) Covariance map and (h) elastic network of LDPV- TLR4 docked complex; Figure S6: A computer-based simulation to model the immune response of antigen and immunoglobulins levels to the BGTV (a), CDPV (b), GMPV (c), and LDPV (d) vaccine candidates without adjuvant, administering three doses over 350 days; Table S1: Nucleotide Sequences of mRNA-Constructed Vaccine Candidates BGTV, CDPV, GMPV, and LDPV for *T. rubrum*; Table S2: Refined Structures Obtained for BGTV, CDPV, GMPV, and LDPV Vaccine Constructs Using GalaxyRefine Server.
