# Correction: Elalouf et al. Bioinformatics-Driven mRNA-Based Vaccine Design for Controlling Tinea Cruris Induced by *Trichophyton rubrum*. *Pharmaceutics* 2024, *16*, 983

**DOI:** 10.3390/pharmaceutics16101273

**Published:** 2024-09-29

**Authors:** Amir Elalouf, Hanan Maoz, Amit Yaniv Rosenfeld

**Affiliations:** Department of Management, Bar-Ilan University, Ramat Gan 5290002, Israel; hanan.maoz@gmail.com (H.M.); amityaro1@gmail.com (A.Y.R.)

In the original publication [1], there was a mistake in uploading Figure 9. As a result, the current online version of the article contains identical images of Figures 8 and 9. We replaced Figure 9 with the correct image below to rectify this error. The email addresses of the other two authors have also been corrected. The authors state that the scientific conclusions are unaffected. This correction was approved by the Academic Editor. The original publication has also been updated.

## Figures and Tables

**Figure 9:**
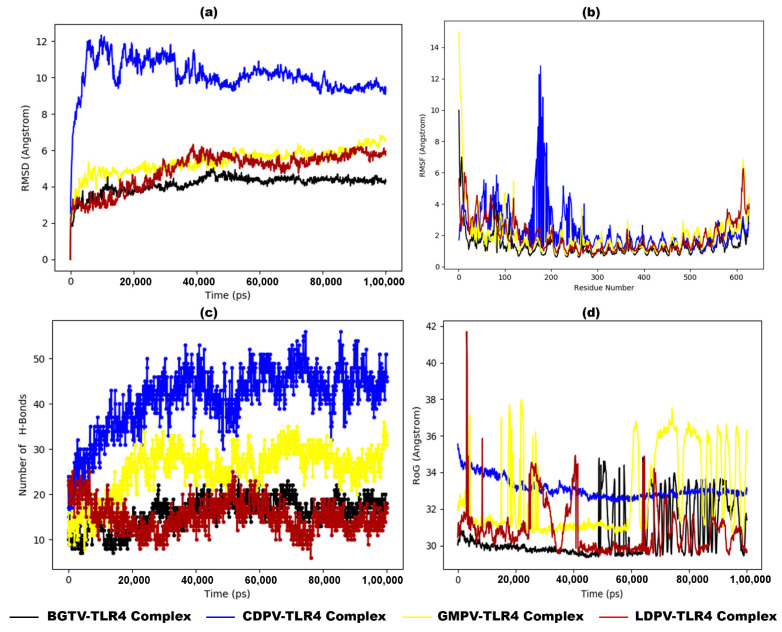
MD simulation results of dock complexes of potential vaccine candidates (BGTV (black), CDPV (blue), GMPV (yellow), and LDPV (red)) with TLR4 backbone. (**a**) Trajectory analysis of the RMSD between C-alpha atoms of dock complexes over time, (**b**) RMSF plot, (**c**) number of hydrogen bond formations, and (**d**) radius of gyration (RoG) plot.
