# Supplementary material for: Bioinformatics-Driven mRNA-Based Vaccine Design for Controlling Tinea Cruris Induced by Trichophyton rubrum
Source: Pharmaceutics. 2024 Jul 25;16(8):983. doi: 10.3390/pharmaceutics16080983 (PMC11357599; doi:10.3390/pharmaceutics16080983)
Supplement: Supplementary file 1 [file pharmaceutics-16-00983-s001.zip › pharmaceutics-3000129-supplementary.pdf]

Supplementary Materials

Bioinformatics-Driven mRNA-Based Vaccine Design for Controlling Tinea Cruris Induced by *Trichophyton rubrum*

Amir Elalouf *, Hanan Maoz and Amit Yaniv Rosenfeld

* **Correspondence:** amir.elalouf@biu.ac.il; Tel.: +972-3-5317128

1. **Supplementary Figures**

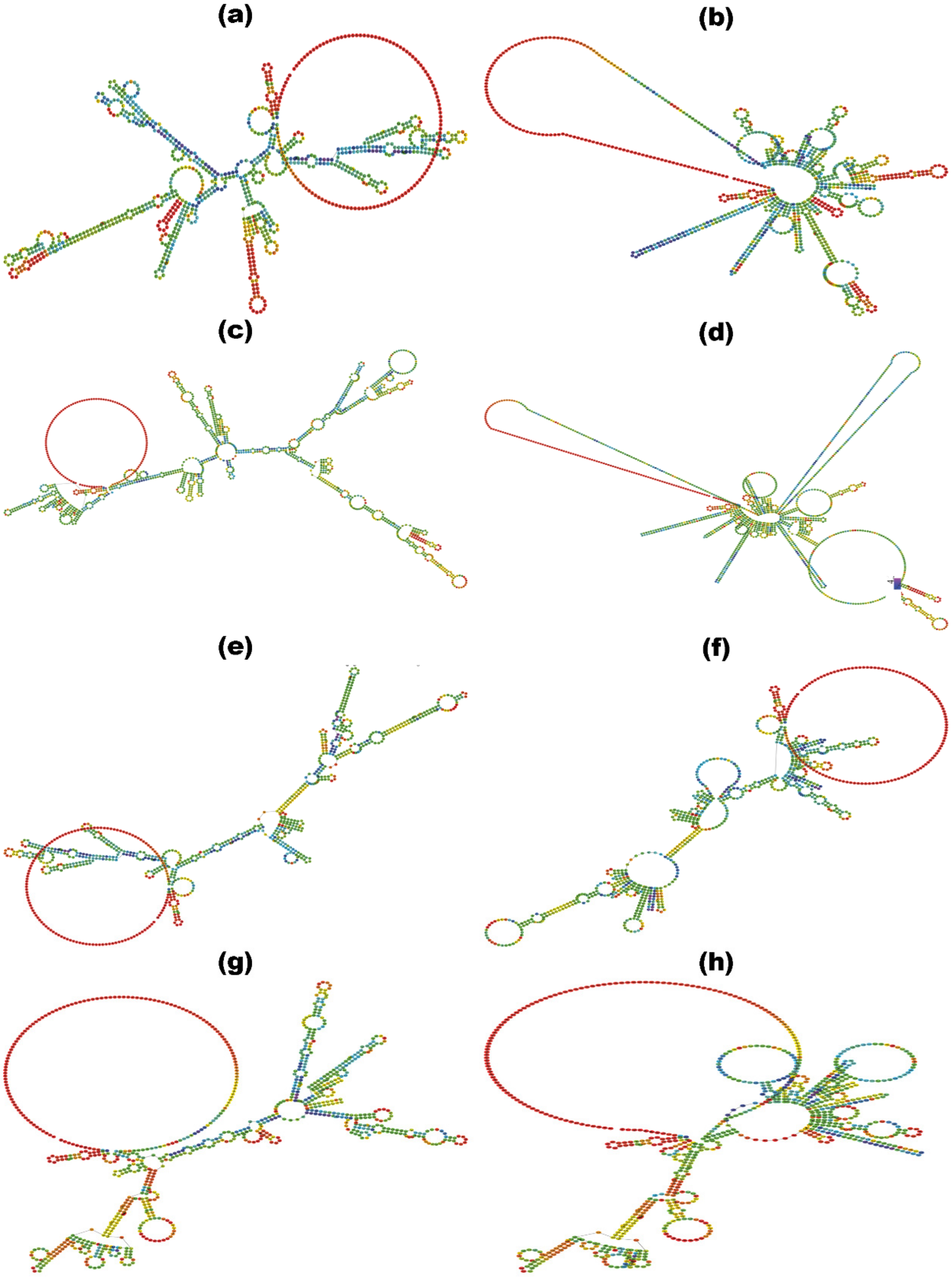

Supplementary Figure S1. Revealing the minimal free energy (MFE) and stability of optimal secondary structures of mRNA BGTV (MFE (a) and Centroid (b)), CDPV (MFE (c) and Centroid (d)), GMPV (MFE (e) and Centroid (f)), and LDPV (MFE (g) and Centroid (h)) candidates.

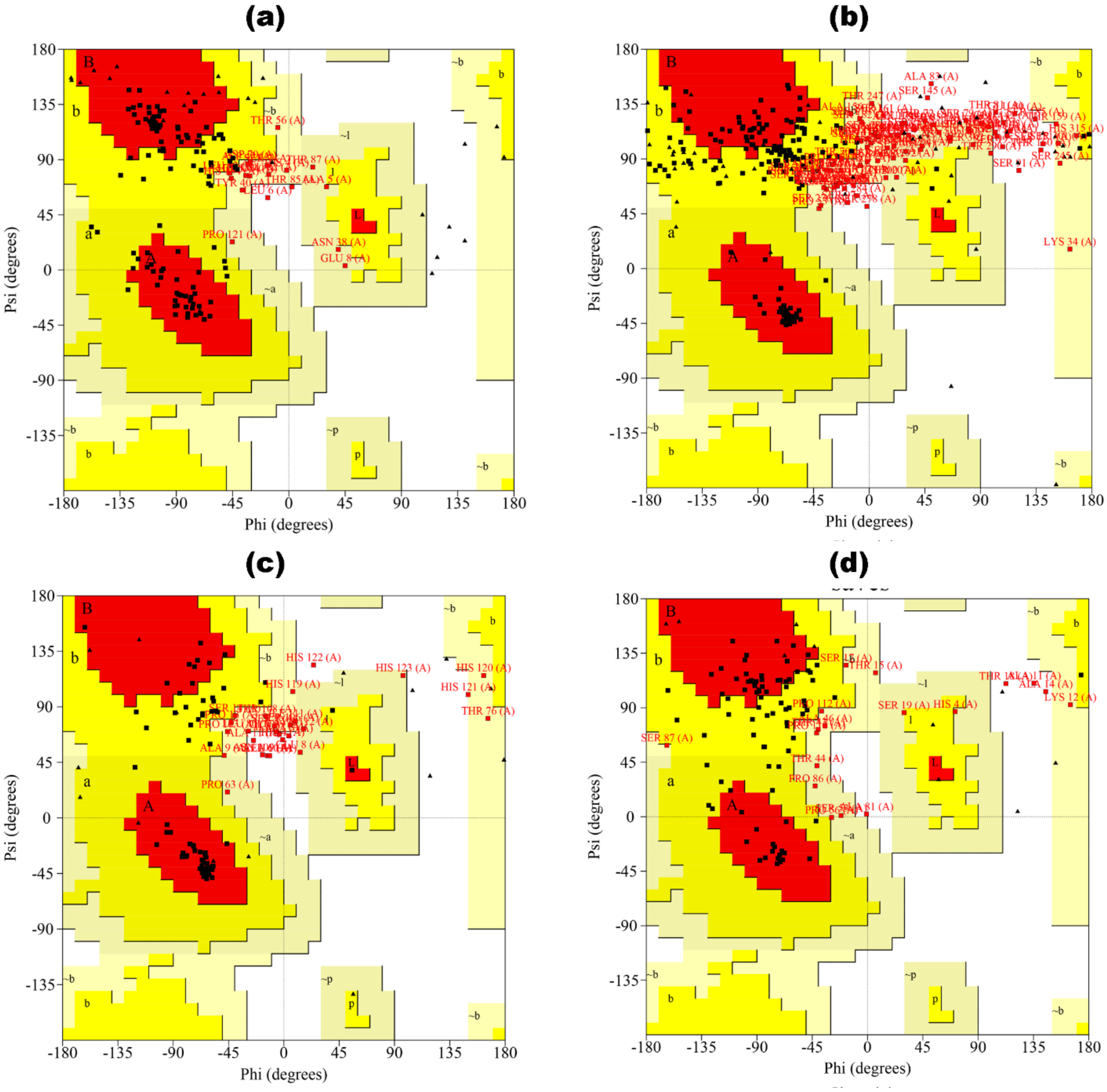

Supplementary Figure S2. Ramachandran plots of 3D modeled structures of vaccines, i.e., BGTV (a), CDPV (b), GMPV (c), and LDPV (d).

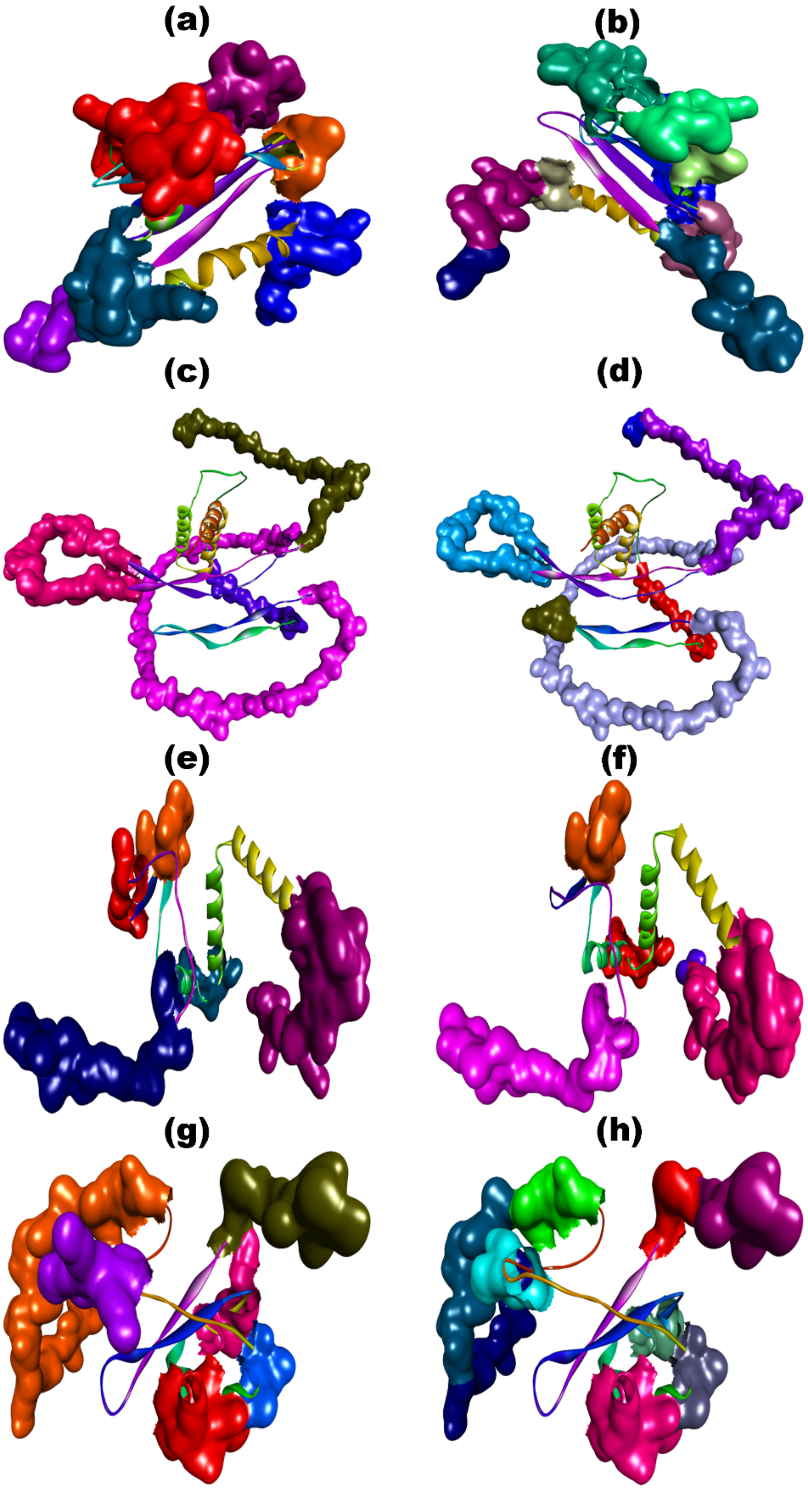

Supplementary Figure S3. Spatial Distribution of Continuous and Discontinuous B-cell Epitopes in BGTV, CDPV, GMPV, and LDPV. Continuous (a) and Discontinuous (b) B-cell Epitopes of BGTV are shown in different color surfaces. Continuous (c) and Discontinuous (d) B-cell Epitopes of CDPV are shown in different color surfaces. Continuous (e)

and Discontinuous (f) B-cell Epitopes of GMPV are shown in different color surfaces. Continuous (g) and Discontinuous (h) B-cell Epitopes of LDPV are shown in different color surfaces.

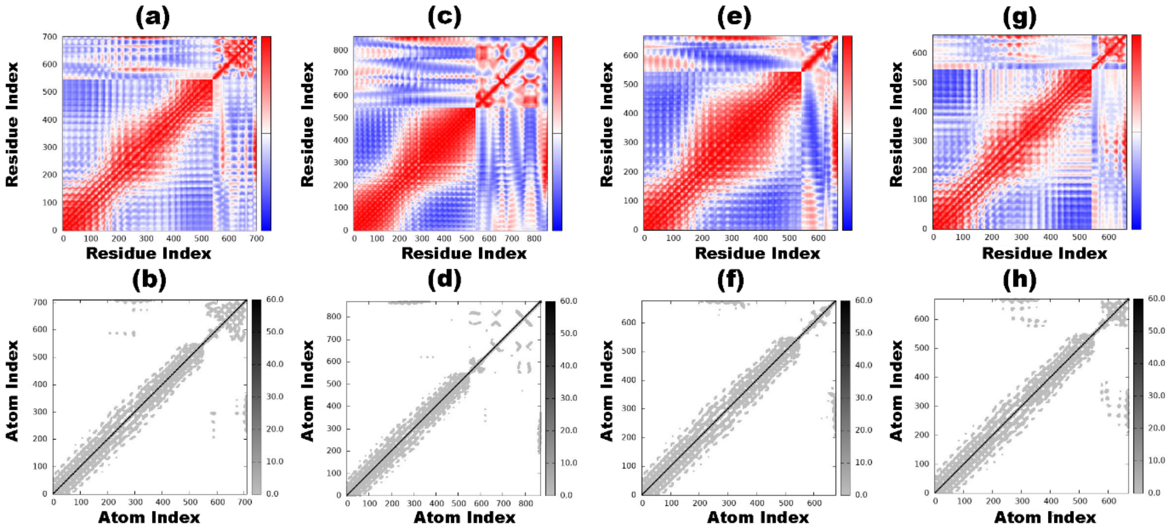

Supplementary Figure S4. Covariance and elastic network maps of vaccine candidates against *T. rubrum* and TLR2 receptor complexes by iMODs. (a) Covariance map and (b) elastic network of BGTV- TLR2; (c) Covariance map and (d) elastic network of CDPV - TLR2; (e) Covariance map and (f) elastic network of GMPV - TLR2; (g) Covariance map and (h) elastic network of LDPV- TLR2 docked complex.

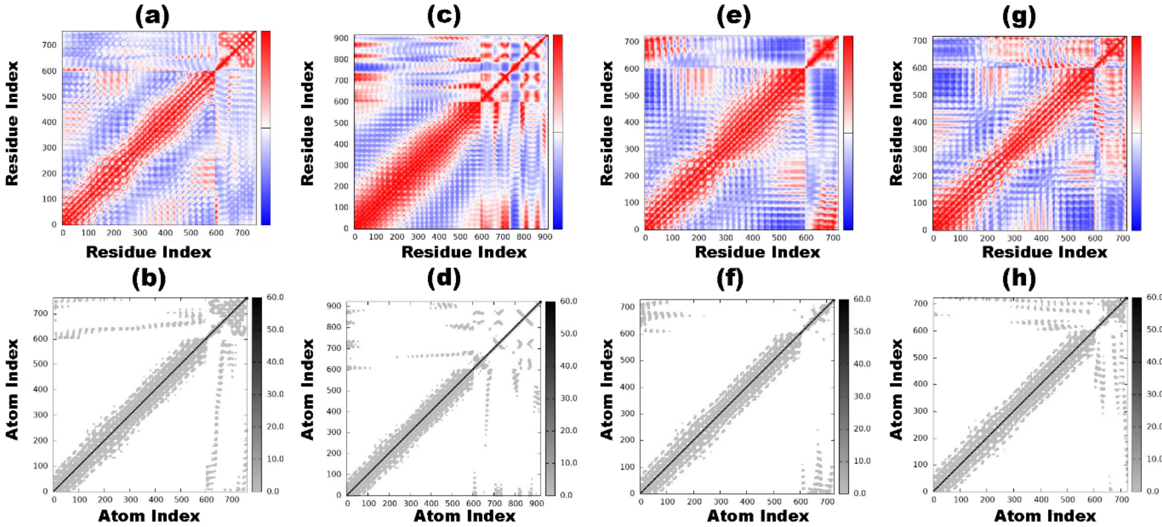

Supplementary Figure S5. Covariance and elastic network maps of vaccine candidates against *T. rubrum* and TLR4 receptor complexes by iMODs. (a) Covariance map and (b) elastic network of BGTV- TLR4; (c) Covariance map and (d) elastic network of CDPV – TLR4; (e) Covariance map and (f) elastic network of GMPV – TLR4; (g) Covariance map and (h) elastic network of LDPV- TLR4 docked complex.

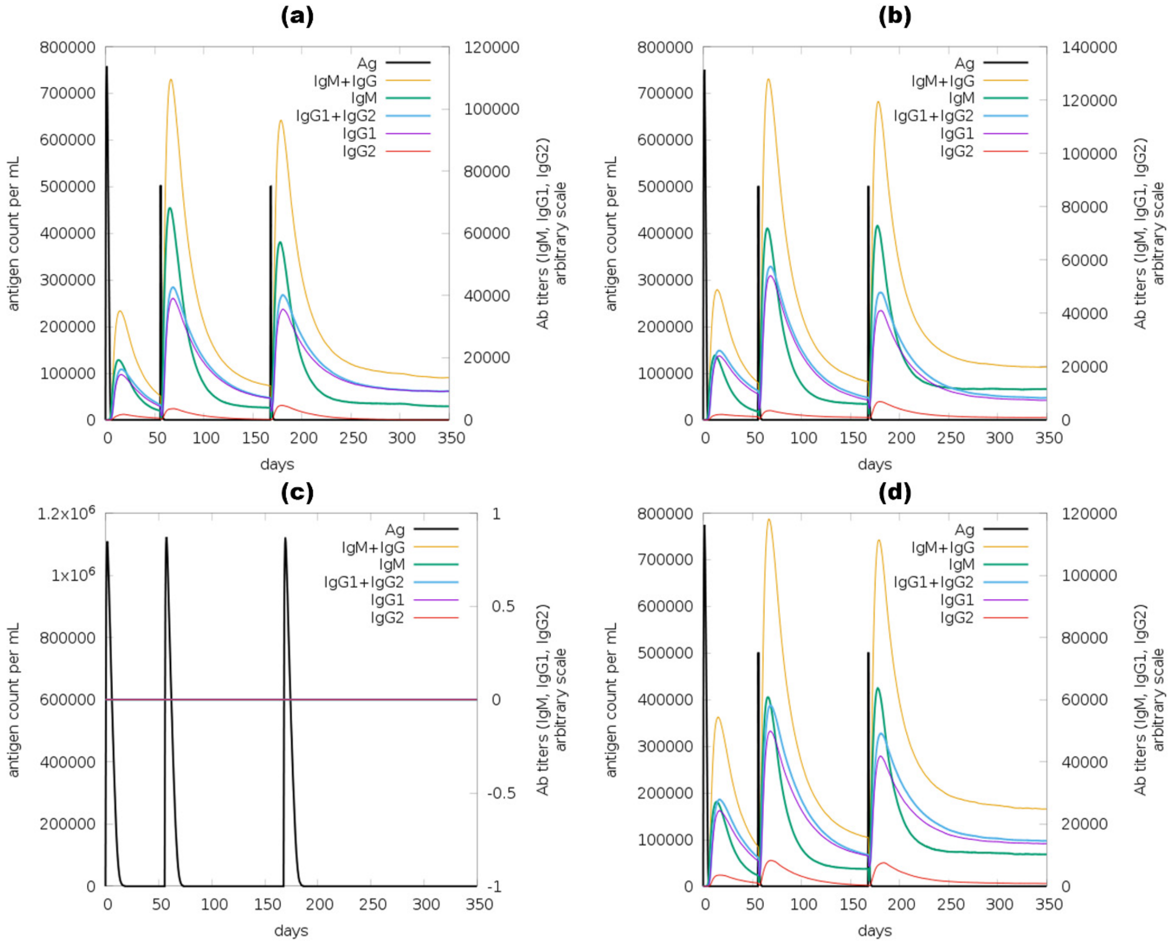

Supplementary Figure S6. A computer-based simulation to model the immune response of antigen and immunoglobulins levels to the BGTV (a), CDPV (b), GMPV (c), and LDPV (d) vaccine candidates without adjuvant, administering three doses over 350 days.

2. Supplementary Tables

Supplementary Table S1. Nucleotide Sequences of mRNA-Constructed Vaccine Candidates BGTV, CDPV, GMPV, and LDPV for *T. rubrum*.

Vaccines	Nucleotide sequences of mRNA Vaccine Candidates
**BGTV**	m7GpppmACAUUUGCUUCUGACACAACUGUGUUCACUAGCAACCUCAAACAGACAC<br>CGCCGCCAUGAUGGACGCCAUGAAGAGGGGCCUGUGCUGCGUGCUGCUGCUGUGC<br>GGCGCCGUGUUCGUGAGCCCCGCUCCGCCGCACGCUCUGUCUGAAGCUGCUGCUAA<br>AAACGAAGUUCAGCCGCGUAUGUUCACCGAAGUUCAGGCUCUGUACGGUGACAAA<br>AUGAAAAAAACCUCUGCUGACAACUCUUACCAGGACCCGCUGGCUGACGUUAAAUC<br>UGCUGCUUACUCUAACGGUACCGAAUUCUUCAUGAAAGGUCCGGGUCCGGGUUCU<br>UACCAGGACCCGCUGGCUGACGUUGGUCCGGGUCCGGGUCAGGAACUGCAGACCAA<br>CACCAUCCGUGUUGGUCCGGGUCCGGGUUACACCCGUUACACCUCUGUUAUCGACG<br>GUCCGGGUCCGGGUUACACCAACGUUAUCGGUUUCUUCGCUGGUGGGCCGGGGCC

	GGGCUUCUGGGGUUACAAUAUCUACUCUUGGGGUCCGGGUCCGGGUAACUUCAAC<br>GUUCCGGUUUUCUUCGCUGGUCCGGGUCCGGGUCACCACCACCACCACCACGCUCG<br>CUUUCUUGCUGUCCAAUUUCUAUUAAAGGUUCCUUUGUUCCGUAAGUCCAACUAC<br>UAAACUGGGGGAUAUUAUGAAGGGCCUUGAGCAUCUGGAUUCUGCCUAAUAAAAA<br>ACAUUUAUUUUCAUUGCGCUCGCUUUCUUGCUGUCCAAUUUCUAUUAAAGGUUCC<br>UUUGUUCCGUAAGUCCAACUACUAAACUGGGGGAUAUUAUGAAGGGCCUUGAGCA<br>UCUGGAUUCUGCCUAAUAAAAAACAUUUAUUUUCAUUGCAAAAAAAAAAAAAAAA<br>AAAAAAAAAAAAAAAAAAAAAAAAAAAAAAAAAAAAAAAAAAAAAAAAAAAAAAAAA<br>AAAAAAAAAAAAAAAAAAAAAAAAAAAAAAAAAAAAAAAAAAAAAAA
**CDPV**	m7GpppmACAUUUGCUUCUGACACAACUGUGUUCACUAGCAACCUCAAACAGACAC<br>CGCCGCCAUGAUGGACGCCAUGAAGAGGGGCCUGUGCUGCGUGCUGCUGCUGUGC<br>GGCGCCGUGUUCGUGAGCCCCGCUCCGCCGCACGCUCUGUCUGAAGCUGCUGCUAA<br>AUGCUCUAACGCUGACUUCCAGCACGGUCUGCGUGACUGCACCCACGAAGCUUGCC<br>CGGGUGAAAAAGUUGAACAGGUUGUUCAGGCUGGUCUGCAGGCUUGCCGUGAAA<br>UGGGUGGUGCUCCGGGUUCUUCUACCGGUGCUCCGACCACCGGUACCGGUUCUGG<br>UACCACCACCGGUACCCCGACCUCUGGUUCUGGUUCUGAAACCACCGCUCCGUCUA<br>CCUCUGGUUCUGGUUCUGCUCCGGCUCCGACCUCUGGUGGUCACUCUACCCCGUAC<br>UCUACCAUCCCGGCUGGUCCGACCGUUAUCACCUCUGGUACCCACGUUGUUACCAC<br>CUCUCGUCCGCCGACCACCCUGUACACCGAAGUUUCUGGUUCUCAGACCGGUUCUG<br>AAUCUUCUUCUCCGACCGGUACCGGUUCUGAAUCUACCUCUGCUCCGGAAACCACC<br>UCUCCGUCUUCUACCGAAGGUGGUUCUUCUCCGUCUUCUACCGAAGGUUCUGGUA<br>ACGGUGGUUCUGGUGGUUCUGAAACCUCUGGUUCUGGUAACGGUCCGUCUCAGAC<br>CCCGUCUCAGGGUAUCGCUCCGAAAGCUACCGGUCUGGGUGUUAAAAAGUUGUU<br>ACCACCUCUCGUCCGCCGACCACCCUGUACACCGAAGUUUCUGGUUCUCAGACCGC<br>UGCUUACACCUCUGGUUCUGGUAACGGUCCGUCUCAGACCCCGUCUCAGGGUGGU<br>AUCGCUCCGGCUGCUUACACCCACGUUGUUACCACCUCUCGUCCGCCGACCACCCUG<br>UACACCGAAGUUUCUGGUUCUGGUCCGGGUCCGGGUUCUUCUUCUCCGACCGGUA<br>CCGGUUCUGAAUCUGGUCCGGGUCCGGGUCCGUCUUCUACCGAAGGUGGUUCUUC<br>UGGUCCGGGUCCGGGUCACCACCACCACCACCACGCUCGCUUUCUUGCUGUCCAAU<br>UUCUAUUAAAGGUUCCUUUGUUCCGUAAGUCCAACUACUAAACUGGGGGAUAUUA<br>UGAAGGGCCUUGAGCAUCUGGAUUCUGCCUAAUAAAAAACAUUUAUUUUCAUUGC<br>GCUCGCUUUCUUGCUGUCCAAUUUCUAUUAAAGGUUCCUUUGUUCCGUAAGUCCA<br>ACUACUAAACUGGGGGAUAUUAUGAAGGGCCUUGAGCAUCUGGAUUCUGCCUAAU<br>AAAAAACAUUUAUUUUCAUUGCAAAAAAAAAAAAAAAAAAAAAAAAAAAAAAAAAA<br>AAAAAAAAAAAAAAAAAAAAAAAAAAAAAAAAAAAAAAAAAAAAAAAAAAAAAAAAA<br>AAAAAAAAAAAAAAAAAAAAAAAAAAAAA
**GMPV**	m7GpppmACAUUUGCUUCUGACACAACUGUGUUCACUAGCAACCUCAAACAGACAC<br>CGCCGCCAUGAUGGACGCCAUGAAGAGGGGCCUGUGCUGCGUGCUGCUGCUGUGC<br>GGCGCCGUGUUCGUGAGCCCCGCUCCGCCGCACGCUCUGUCUGAAGCUGCUGCUAA<br>ACCGUCUACCUUCUCUUCUGUUCCGGAAGCUAUCGGUGACCUGGACCCGAUCUCU<br>GCUUCUAUCGAAGGUCUGUCUCAGCGUAUCGCUCAGUCUCCGGGUGGUAUCACCG<br>AACUGAUGUCUAAAAAACUGUCUCAGCGUAUCGCUCAGUCUCCGGGUGGUAUCAC<br>CGAACUGGCUGCUUACGCUACCUCUACCAAAGUUCCGCUGAUCAAAGCUGUUCCGG<br>GUGGUGCUGCUUACGCUCAGUCUCCGGGUGGUAUCACCGAAGGUCCGGGUCCGGG

	UAUGUCUGUUACCAACGACAUCUACGACGGUCCGGGUCCGGGUCACCACCACCACC<br>ACCACGCUCGCUUUCUUGCUGUCCAAUUUCUAUUAAAGGUUCCUUUGUUCCGUAA<br>GUCCAACUACUAAACUGGGGGAUAUUAUGAAGGGCCUUGAGCAUCUGGAUUCUGC<br>CUAAUAAAAAACAUUUAUUUUCAUUGCGCUCGCUUUCUUGCUGUCCAAUUUCUAU<br>UAAAGGUUCCUUUGUUCCGUAAGUCCAACUACUAAACUGGGGGAUAUUAUGAAGG<br>GCCUUGAGCAUCUGGAUUCUGCCUAAUAAAAAACAUUUAUUUUCAUUGCAAAAAA<br>AAAAAAAAAAAAAAAAAAAAAAAAAAAAAAAAAAAAAAAAAAAAAAAAAAAAAAAAA<br>AAAAAAAAAAAAAAAAAAAAAAAAAAAAAAAAAAAAAAAAAAAAAAAAAAAAAAAAA
**LDPV**	m7GpppmACAUUUGCUUCUGACACAACUGUGUUCACUAGCAACCUCAAACAGACAC<br>CGCCGCCAUGAUGGACGCCAUGAAGAGGGGCCUGUGCUGCGUGCUGCUGCUGUGC<br>GGCGCCGUGUUCGUGAGCCCCGCUCCGCCGCACGCUCUGUCUGAAGCUGCUGCUAA<br>AGGUGCUACCAUCUCUACCUCUAUGCCGAUGCCGACCCCGUCUGGUCCGCAGCCGC<br>AAAUGCCAGGUAUCGUAAGCAACUGCAAAAAAACCACCACCCGUGCUAUGACCACCA<br>CCAUCUCUUCUGACGCUCCGGCUGCUUACUCUAUCCAGACCAAAUACGGUAUCUCU<br>ACCGACCAGUUCAAAGCUUGGAACCCGUACAUCAACGCUGAAGCUGCUUACCCGUC<br>UACCACCACCACCGCUAAACCGGGUCCGGGUCCGGGUACCCGUGCUAUGACCACCAC<br>CAUCUCUGGUCCGGGUCCGGGUCACCACCACCACCACCACGCUCGCUUUCUUGCUG<br>UCCAAUUUCUAUUAAAGGUUCCUUUGUUCCGUAAGUCCAACUACUAAACUGGGGG<br>AUAUUAUGAAGGGCCUUGAGCAUCUGGAUUCUGCCUAAUAAAAAACAUUUAUUUU<br>CAUUGCGCUCGCUUUCUUGCUGUCCAAUUUCUAUUAAAGGUUCCUUUGUUCCGUA<br>AGUCCAACUACUAAACUGGGGGAUAUUAUGAAGGGCCUUGAGCAUCUGGAUUCUG<br>CCUAAUAAAAAACAUUUAUUUUCAUUGCAAAAAAAAAAAAAAAAAAAAAAAAAAAA<br>AAAAAAAAAAAAAAAAAAAAAAAAAAAAAAAAAAAAAAAAAAAAAAAAAAAAAAAAA<br>AAAAAAAAAAAAAAAAAAAAAAAAAAAAAAAAAAA

Supplementary Table S2. Refined Structures Obtained for BGTV, CDPV, GMPV, and LDPV Vaccine Constructs Using GalaxyRefine Server.

Vaccines	GDT-HA	RMSD	MolProbity	Clash score	Poor rotamers	Rama favored
**BGTV**	0.8671	0.649	1.093	3	0	98.7
**CDPV**	0.8221	0.791	1.373	2.4	0	95
**GMPV**	0.8669	0.741	1.325	4.6	0	97.5
**LDPV**	0.8845	0.642	1.285	1.8	0	94.9
